# Characterization of Prurigo Nodules in Adults With Moderate‐To‐Severe Atopic Dermatitis in Japan: A 2‐Year Observational Study

**DOI:** 10.1111/1346-8138.70345

**Published:** 2026-07-02

**Authors:** Yoko Kataoka, Takafumi Etoh, Hidehisa Saeki, Norito Katoh, Satoshi Teramukai, Yuki Tajima, Kwinten Bosman, Marius Ardeleanu, Kazuhiko Arima

**Affiliations:** ^1^ Department of Dermatology Osaka Habikino Medical Center Osaka Japan; ^2^ Atago Dermatology Clinic Tokyo Japan; ^3^ Department of Dermatology Tokyo Teishin Hospital Tokyo Japan; ^4^ Department of Dermatology Nippon Medical School Tokyo Japan; ^5^ North Campus Kyoto Prefectural University of Medicine Kyoto Japan; ^6^ Department of Biostatistics Kyoto Prefectural University of Medicine Kyoto Japan; ^7^ Sanofi K.K Tokyo Japan; ^8^ Sanofi Cambridge Massachusetts USA; ^9^ Regeneron Pharmaceuticals, Inc. Tarrytown New York USA

**Keywords:** atopic dermatitis, Japan, observational study, prurigo, symptom burden

## Abstract

Clinical phenotypes of atopic dermatitis (AD) can vary, with some individuals displaying difficult‐to‐treat prurigo nodularis‐like lesions. We aimed to assess the real‐world disease burden and treatment course of Japanese adults with AD‐associated prurigo nodules (AD‐Pn) receiving conventional AD treatments. Of the 300 participants with moderate‐to‐severe AD registered in the observational ADDRESS‐J study between July 29, 2016 and July 4, 2017 (before the first biologic for AD was introduced), 285 had baseline and post‐baseline prurigo assessments and were included in this exploratory analysis (108 with AD‐Pn and 177 without Pn). Participants with AD‐Pn versus those without Pn included more males (73.1% vs. 53.1%), and had more mean ± standard deviation (SD) visits for AD treatment in the previous year (11.5 ± 16.1 vs. 8.7 ± 6.6 visits), a higher proportion with an Investigator's Global Assessment (IGA) 4 score (39.9% vs. 18.6%), a higher mean ± SD Eczema Area and Severity Index (EASI) score (28.4 ± 15.1 vs. 23.5 ± 15.5), and a higher mean ± SD Patient‐Oriented Eczema Measure (POEM) score (18.8 ± 6.2 vs. 15.6 ± 6.8). At baseline, 14.8% and 7.4% of participants with AD‐Pn had been treated with oral immunosuppressants and oral corticosteroids, respectively, compared with 8.5% and 2.3%, respectively, of those without Pn. Individuals with AD‐Pn previously received more intensive topical treatments. At 2 years, participants with AD‐Pn had more severe AD than those without Pn, as assessed by IGA 4 scores (9.1% vs. 0.9%), and higher mean ± SD EASI total scores (12.5 ± 10.7 vs. 7.0 ± 8.1). In summary, individuals with AD‐Pn were mostly males, whose AD was more severe and had improved less than those without Pn while receiving mostly conventional AD treatments over 2 years. AD‐Pn might be an indicator for targeted treatment to improve long‐term AD outcomes.

**Clinical Trial Registration:** ADDRESS‐J (UMIN Clinical Trial Registration: UMIN000022623)

## 1 Introduction

The clinical phenotypes of atopic dermatitis (AD) can vary, and some individuals display prurigo nodularis (PN)‐like lesions [1, 2, 3]. A systematic review and meta‐analysis of > 100 studies of AD showed that common morphologies included follicular (34%), papular lichenoid (22%), nummular (13%), and prurigo lesions (7%) [4]. PN is a cutaneous disease that is distinct from AD, but prurigo nodules (Pn) can occur secondary to AD (i.e., AD‐associated Pn [AD‐Pn]). Pn present mainly on the extremities as single to multiple excoriated hyperkeratotic, intensely itchy papules and nodules. AD‐Pn more commonly affects adults and individuals of South‐East Asian or African origin [1]. Studies from Singapore and Thailand have reported a higher prevalence of AD‐Pn compared with European prevalence data (27% vs. 4%, respectively) [4]. We have previously reported, in our AD registry study performed in Japan, a high prevalence of AD‐Pn in participants with moderate‐to‐severe AD (30.9% in participants with moderate AD and 56.3% in those with severe AD) [2].

AD‐Pn has traditionally been considered to consist of refractory AD skin eruptions, or a phenotype of AD that is often difficult to treat. Retrospective, single‐center data have described the prevalence, disease burden, and epidemiology of individuals with AD‐Pn in Japan [5, 6]. A 2014 Japanese university hospital‐based study reported that among 257 individuals with AD, 33 (12.8%) were diagnosed with AD‐Pn [6]. However, there is scant evidence on the long‐term prognosis of individuals with AD‐Pn, particularly with regard to how the extent of Pn relates to disease severity, mainly because there are very few prospective cohort studies [7]. The impact of AD‐Pn on overall AD management, therefore, remains unclear [8, 9]. Access to such information may help clinicians to accurately identify individuals with severe prurigo as appropriate candidates for systemic treatment [7].

AD‐Pn also deserves close attention in terms of assessing the severity of skin symptoms. For severity assessments of AD, well‐validated instruments are used for measuring signs and symptoms of AD, such as the Eczema Area and Severity Index (EASI) score [10] and the Severity Scoring of Atopic Dermatitis (SCORAD) index [11], to grade the intensity of lesions. These usually assess the disease severity by the area of skin lesions and the degree of erythema, papules, excoriations, and lichenification. However, these measurements do not easily capture the severity of AD‐Pn, which may affect a smaller body surface area (BSA) by themselves, and the morphology does not match each component of the skin lesions to be assessed. Thus, established measurements for AD cannot specifically assess the severity of AD‐Pn [8, 9].

The pathomechanism of PN is becoming better understood and the molecular differences between PN and AD‐Pn have been clarified [1, 12, 13, 14, 15]. Moreover, molecularly targeted medicines have shown marked effects on both previously treatment‐refractory PN and AD‐Pn [16, 17, 18]. A real‐world, prospective cohort observation of AD‐Pn may have significance for predicting resistance to treatment and selecting the appropriate drug for individuals with AD‐Pn who require such novel therapies.

ADDRESS‐J was a 2‐year, noninterventional, observational registry study of adult Japanese individuals with moderate‐to‐severe AD (Investigator's Global Assessment [IGA] score 3 or 4), which reported that approximately half of the enrolled participants were inadequately controlled under conventional treatment before biologics, suggesting a need for treatment escalation [2, 19]. The present *post hoc* exploratory analysis assessed the real‐world disease burden and treatment course of individuals with AD‐Pn in the ADDRESS‐J Registry. We characterized the baseline disease burden of individuals with AD‐Pn and their progression up to 2 years.

## 2 Methods

### 2.1 Study Design

ADDRESS‐J was a 2‐year prospective, noninterventional, longitudinal, observational study (UMIN‐Clinical Trial Registry: UMIN000022623); details of the study design have been reported previously [2]. In brief, the study included individuals with moderate‐to‐severe AD from 30 medical institutions throughout Japan who were undergoing treatment escalation at their first visit (baseline). The primary objective was to infer the rate of AD flare, whereas the secondary objectives included exploratory analyses with a pre‐specified descriptive analysis plan; all the analyses presented in this manuscript are exploratory [2]. This included 10 hospitals and 20 clinics in Japan (hospitals were defined as a medical institute with ≥ 20 beds for admission; none of the study clinics had beds available for admission).

#### 2.1.1 Study Population

Inclusion criteria were specified as an Investigator's Global Assessment (IGA) score ≥ 3 (moderate‐to‐severe atopic dermatitis [AD]), suggesting inadequate disease control under current treatment, and participants were required to have treatment escalation at their baseline visit. Treatment escalation included initiation of any of the following treatments: a new AD medication dosage (topical/oral corticosteroids or topical/oral immunosuppressants); change to a higher‐potency class of topical corticosteroids (TCS); or an increase of AD medication dosage (topical/oral corticosteroids or topical/oral immunosuppressants). For the purposes of this study, ultraviolet phototherapy and antihistamine/anti‐allergic drugs were not considered as an escalation of treatment at baseline. Another aspect considered to be an escalation of treatment included thorough implementation of patient education intended as standard of care for AD (topical/oral corticosteroids or topical/oral immunosuppressants); this did not include brief instructions on treatment usually provided on a routine outpatient basis. Exclusion criteria were treatment with an investigational drug within 8 weeks before the baseline visit or any skin comorbidities that could potentially interfere with study assessments.

All individuals satisfying the prespecified inclusion criteria who were willing and able to comply with study procedures for a 2‐year observation period and who provided signed informed consent were enrolled into the study until the target enrolment of 300 participants was met.

### 2.2 Ethical Considerations

The study protocol and other necessary documents were submitted to and approved by all relevant institutional review board and ethics committees prior to enrollment of the first participant. The study was conducted according to the ethical principles defined by the 18th World Medical Association General Assembly Declaration of Helsinki and its amendments, as well as the Ethical Guidelines for Medical and Health Research Involving Human Subjects (established on December 22, 2014). All participants completed written informed consent forms before initiating any study procedure.

### 2.3 Objectives of Present Analyses

The objectives of this *post hoc* exploratory analysis were to describe demographics and baseline severity of adults with moderate‐to‐severe AD by Pn presence, and to describe the clinical course of Pn appearing in these individuals over the entire study period (maximum 2 years) with conventional treatments for AD. An exploratory objective was the development of a method of severity classification for individuals with AD‐Pn (based on number and size of nodules) that may serve as a framework for evaluation of treatment response.

### 2.4 Data Collection and Assessments

Clinical observations and assessments were performed at baseline (Day 1) and every 3 months thereafter; details of data collected at baseline have been previously reported [2]. Baseline data collection included demographics (sex, age, height, weight, education, and employment classification), disease characteristics (AD duration and number of clinic/hospital visits for AD treatments in the previous year), and medical history of other allergic diseases (e.g., family history). Post‐baseline assessments, including clinical observation and assessments of the current treatment (i.e., effectiveness and safety measures), were collected approximately every 3 months [19].

Clinical and patient‐reported outcomes (PROs) of AD signs and symptoms collected at baseline included IGA, EASI, percent body surface area (BSA) affected, Dermatology Life Quality Index (DLQI), EuroQOL group health questionnaire with five dimensions (EQ‐5D; questionnaire for mobility, self‐care, usual activity, pain/discomfort, and anxiety/depression) and utility score, EQ visual analog scale (EQ‐VAS) score for respondent's health status, Patient‐Oriented Eczema Measure (POEM), average Peak Pruritus Numerical Rating Scale (PP‐NRS) maximum itch intensity over the last 7 days, and condition of nodular prurigo. Baseline data also recorded the most recent AD treatments received prior to the baseline visit, including medications and procedures.

Due to the lack of standardized prurigo measures, the study investigators collected the following exploratory data on Pn to perform the exploratory analysis of severity classification: present or absent; the number of Pn (< 10, ≥ 10 to < 100, or ≥ 100); and the size (diameter) of the most significant nodule (< 2, ≥ 2 to < 4, ≥ 4 to < 8, or ≥ 8 mm).

### 2.5 Outcomes

The main study endpoints were assessment of effectiveness of conventional AD treatments by presence or absence of Pn at baseline. Effectiveness measures included the proportion of participants with EASI score change from baseline by 50% and 75%. Other endpoints included change in EASI, percent BSA affected by AD, and PROs, such as average PP‐NRS scores in the previous 7 days [20], and the participant's current health status as determined by DLQI scores [21], POEM scores [22], and utility and EQ‐VAS scores [23]. The following biomarkers were also assessed if available: serum thymus‐ and activation‐regulated chemokine (TARC), peripheral blood eosinophil counts, serum total immunoglobulin E (IgE), and serum lactate dehydrogenase (LDH).

To discern the initial intensity of topical treatment [3], topical corticosteroid (TCS)/topical calcineurin inhibitor (TCI) treatment intensity was individually calculated as accumulated weighted prescribed TCS/TCI dosage for the first 2 weeks inclusive of baseline date adapted from a previous randomized controlled study protocol [24]; each TCS/TCI prescription was weighted as follows: 1 = mild‐potency TCS; 2 = medium‐potency TCS or TCI; 4 = strong‐potency TCS; 8 = very strong‐potency TCS; and 12 = strongest‐potency TCS. The calculated index was expressed in arbitrary units (AU).

### 2.6 Statistical Considerations

The target sample size for the primary analysis was 300 participants, as previously reported [2], however, this did not confer any statistical power for the current *post hoc* comparisons.

To describe the potential differential therapeutic impact of baseline medications/treatments, longitudinal scores for outcome measures were analyzed by use of baseline medications/treatments such as topical medications only, oral nonsteroidal systemic immunosuppressants, oral corticosteroids, and phototherapy. Given the noninterventional nature of the study, these medications/treatments were not necessarily continued for the entire observational period.

The window for the first 3‐month data point was from the day after the baseline visit and up to 4.5 months; the window for subsequent data points was from 1.5 months before to 1.5 months after the prespecified time point. All analyses were descriptive, and no hypothesis was tested. Data analysis was performed using SAS, version 9.2 or later (SAS Institute, Cary, NC, USA).

## 3 Results

### 3.1 Study Population and Baseline Characteristics

A total of 300 participants with moderate‐to‐severe AD were enrolled between July 29, 2016, and July 4, 2017, with the last participant completing the study on July 12, 2019. Twelve individuals were excluded due to nonattendance at post‐baseline visits or lack of responses to PRO questionnaires (Figure S1) [19]. Of the remaining 288 participants, three did not undergo prurigo assessment at baseline. Thus, this *post hoc* exploratory analysis included 285 participants, of whom 239 did not formally withdraw and were classified as retained participants for the duration of the study. In Japan, dupilumab was the first biologic to be marketed for AD in April 2018, and 6 participants were treated with dupilumab only in the later period of observation in this study [19].

**FIGURE 1:**
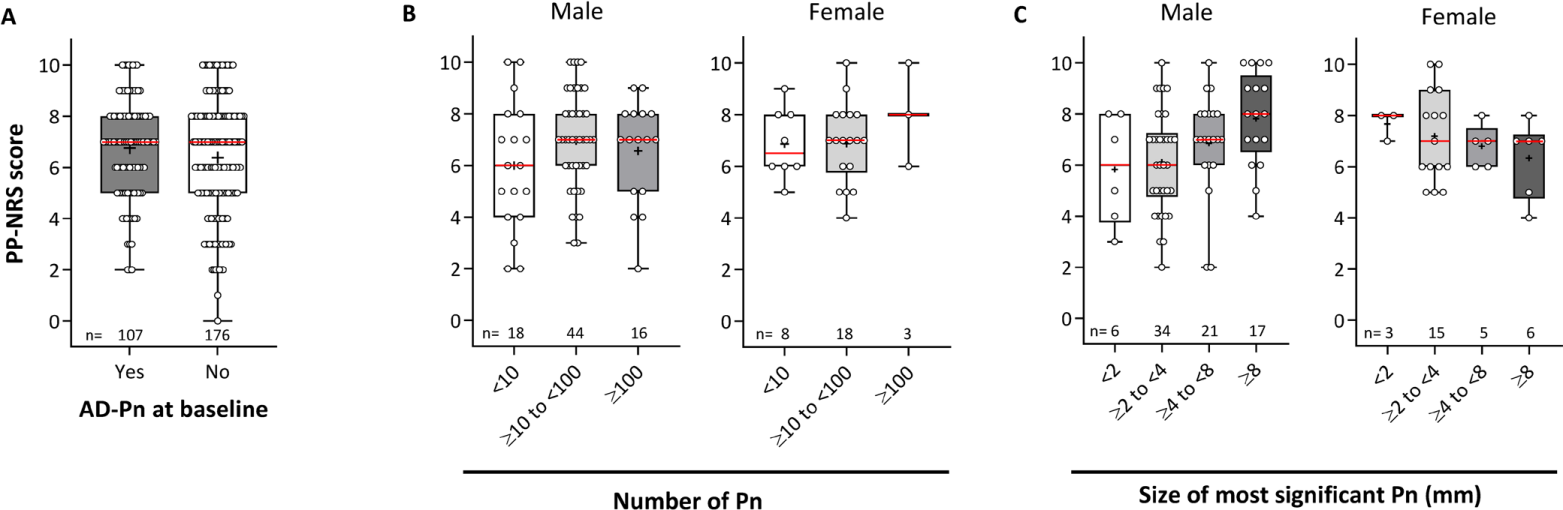
Baseline Peak Pruritus Numerical Rating Scale† scores in participants (A) with and without atopic dermatitis‐associated prurigo nodules, and in males and females by (B) number of prurigo nodules and (C) size of most significant prurigo nodule. †Participants were asked for their average PP‐NRS score in the previous 7 days. AD‐Pn, atopic dermatitis‐associated prurigo nodules; Pn, prurigo nodules; PP‐NRS, Peak Pruritus Numerical Rating Scale.

At baseline, 108 of 285 participants had AD‐Pn and 177 were devoid of Pn (Table 1). Both cohorts with and without Pn had comparable mean ± standard deviation (SD) age, body weight, body mass index, and duration of AD. The cohort with AD‐Pn included a higher proportion of males compared with the cohort without Pn (73.1% vs. 53.1%), and a numerically higher mean ± SD number of visits for AD treatment (11.5 ± 16.1 vs. 8.7 ± 6.6; Table 1). Furthermore, signs and symptoms of AD, as measured by IGA, EASI, BSA, POEM, and PP‐NRS scores, were also numerically higher in participants with AD‐Pn versus those without Pn. In particular, the mean ± SD weekly average PP‐NRS scores at baseline were slightly higher in participants with AD‐Pn versus those without Pn (6.8 ± 1.9 vs. 6.4 ± 2.3); however, among participants registered in hospitals, this trend was not observed (Table 1 and Figure 1A). When analyzed by sex, the mean ± SD baseline weekly average PP‐NRS scores among participants with AD‐Pn versus those without Pn were also numerically higher in both males (6.6 ± 2.1 vs. 5.9 ± 2.1, respectively) and females (7.0 ± 1.5 vs. 6.8 ± 2.4, respectively), and female participants consistently showed higher PP‐NRS scores than male participants irrespective of the presence of AD‐Pn and practice setting.

**TABLE 1:** Summary of baseline demographic and participant characteristics.

Characteristic	AD‐Pn at baseline					
	Yes (*n* = 108)			No (*n* = 177)		
	Overall (*n* = 108)	Hospital (*n* = 59)	Dermatology clinic (*n* = 49)	Overall (*n* = 177)	Hospital (*n* = 74)	Dermatology clinic (*n* = 103)
Age, years	35.9 ± 10.6	34.9 ± 9.8	37.2 ± 11.4	35.2 ± 10.5	33.4 ± 10.6	36.5 ± 10.3
Sex, *n* (%)						
Male	79 (73.1)	46 (78.0)	33 (67.3)	94 (53.1)	33 (44.6)	61 (59.2)
Female	29 (26.9)	13 (22.0)	16 (32.7)	83 (46.9)	41 (55.4)	42 (40.8)
Weight, kg	63.7 ± 11.6	64.2 ± 12.6	63.0 ± 10.3	61.1 ± 11.7	58.4 ± 11.3	63.0 ± 11.7
BMI, kg/m^2^	22.8 ± 3.87	23.0 ± 4.3	22.7 ± 3.4	22.6 ± 3.6	21.8 ± 3.3	23.2 ± 3.7
Age of AD onset, years	8.3 ± 10.8	10.1 ± 12.3	6.1 ± 8.4	9.2 ± 12.7	6.2 ± 10.0	11.4 ± 14.0
AD disease duration, years	27.7 ± 11.9	24.9 ± 11.4	31.1 ± 11.6	26.2 ± 12.1	27.4 ± 11.5	25.3 ± 12.6
No. of visits for AD treatment in previous year	11.5 ± 16.1	13.4 ± 20.6	9.3 ± 6.8	8.7 ± 6.6	7.8 ± 5.5	9.4 ± 7.2
IGA score	3.4 ± 0.5	3.5 ± 0.5	3.3 ± 0.5	3.2 ± 0.4	3.4 ± 0.5	3.0 ± 0.2
EASI score	28.4 ± 15.1	31.5 ± 16.2	24.8 ± 12.9	23.5 ± 15.5	31.5 ± 18.9	17.7 ± 9.0
BSA affected by AD, %	53.9 ± 23.2	57.4 ± 25.0	49.6 ± 20.2	49.0 ± 24.5	61.9 ± 28.0	39.7 ± 16.4
DLQI score	8.2 ± 5.8	9.7 ± 6.7	6.4 ± 4.0	8.3 ± 6.7	11.9 ± 8.2	5.8 ± 3.8
EQ‐5D utility score	0.7779 ± 0.1769	0.7315 ± 0.2030	0.8337 ± 0.1189	0.7935 ± 0.1908	0.6879 ± 0.2252	0.8683 ± 0.1142
EQ‐mobility (Q1): any problems, *n* (%)	16 (14.8)	12 (20.3)	4 (8.2)	28 (15.9)	21 (28.8)	7 (6.8)
EQ‐self‐care (Q2): any problems, *n* (%)	21 (19.4)	14 (23.7)	7 (14.3)	36 (20.5)	21 (28.8)	15 (14.6)
EQ‐usual activity (Q3): any problems, *n* (%)	49 (45.4)	31 (52.5)	18 (36.7)	69 (39.2)	46 (63.0)	23 (22.3)
EQ‐pain/discomfort (Q4): any problems, *n* (%)	91 (84.3)	50 (84.7)	41 (83.7)	136 (77.3)	66 (90.4)	70 (68.0)
EQ‐anxiety/depression (Q5): any problems, *n* (%)	50 (46.3)	34 (57.6)	16 (32.7)	74 (42.0)	49 (67.1)	25 (24.3)
EQ‐VAS	59.2 ± 22.1	55.4 ± 22.8	63.6 ± 20.7	62.6 ± 21.8	52.2 ± 25.3	69.8 ± 15.5
POEM score	18.8 ± 6.2	20.0 ± 6.6	17.4 ± 5.5	15.6 ± 6.8	19.1 ± 6.7	13.2 ± 5.8
Weekly average PP‐NRS score^a^	6.8 ± 1.9	7.1 ± 2.1	6.3 ± 1.7	6.4 ± 2.3	7.3 ± 2.1	5.7 ± 2.2
In males	*n* = 78 6.6 ± 2.1	*n* = 46 7.0 ± 2.2	*n* = 32 6.2 ± 1.7	*n* = 94 5.9 ± 2.1	*n* = 33 6.9 ± 2.0	*n* = 61 5.4 ± 2.0
In females	*n* = 29 7.0 ± 1.5	*n* = 13 7.5 ± 1.3	*n* = 16 6.6 ± 1.5	*n* = 82 6.8 ± 2.4	*n* = 41 7.7 ± 2.2	*n* = 41 6.1 ± 2.4

*Note:* Data are presented as mean ± SD unless stated otherwise.

Abbreviations: AD, atopic dermatitis; AD‐Pn, atopic dermatitis‐associated prurigo nodules; BMI, body mass index; BSA, body surface area; DLQI, Dermatology Life Quality Index; EASI, Eczema Area and Severity Index; EQ, EuroQOL; EQ‐5D, EuroQOL group health questionnaire with five dimensions; IGA, Investigator's Global Assessment; POEM, Patient‐Oriented Eczema Measure; PP‐NRS, Peak Pruritus Numerical Rating Scale; SD, standard deviation; VAS, visual analog scale.

^a^ Participants were asked for their average PP‐NRS score in the previous 7 days (*n* = 107 for Yes and 176 for No).

There were minimal differences between the cohorts with regard to baseline quality of life (QoL) as measured by DLQI and EQ‐5D (Table 1). A detailed investigation of the anxiety/depression domain of the EQ‐5D also demonstrated an overall similar distribution of burden in the two cohorts, but participants in hospitals were affected by more severe problems than those presenting to dermatology clinics (Table S1).

### 3.2 Baseline Medications and Therapies

At baseline, nearly all the participants were receiving topical AD therapy (284/285, 99.7%), with the most common topical treatment being very strong‐potency TCS (246/285, 86.5%), followed by medium‐potency TCS (143/285, 50.3%; Table 2). The use of the strongest‐potency TCS, strong‐potency TCS, and TCI was higher among those with AD‐Pn (45.4%, 28.7%, and 40.7%, respectively) than in those without Pn (39.0%, 20.9%, and 35.0%, respectively).

**TABLE 2:** Baseline atopic dermatitis medications and therapies.

Type of concomitant medication, *n* (%)	AD‐Pn at baseline	
	Yes (*n* = 108)	No (*n* = 177)
Topical		
Any TCS/TCI^a,^, ^b^	108 (100.0)	176 (99.4)
TCS: strongest	49 (45.4)	69 (39.0)
TCS: very strong	93 (86.1)	153 (86.4)
TCS: strong	31 (28.7)	37 (20.9)
TCS: medium	51 (47.2)	92 (52.0)
TCS: weak	2 (1.9)	6 (3.4)
TCI	44 (40.7)	62 (35.0)
Topical only	79 (73.1)	141 (79.7)
Systemic anti‐inflammatory		
Any oral	24 (18.5)	18 (10.2)
Oral corticosteroids	8 (7.4)	4 (2.3)
Oral immunosuppressants	16 (14.8)	15 (8.5)
Biologics^c^	—	—
UV phototherapy	6 (5.6)	10 (5.6)
Adjunctive		
Antihistamines/anti‐allergic drugs^d^	87 (80.6)	146 (82.5)
Chinese herbal medicine	3 (2.8)	3 (1.7)
Psychosomatic approach	—	—

Abbreviations: AD‐Pn, atopic dermatitis‐associated prurigo nodules; TCI, topical calcineurin inhibitor; TCS, topical corticosteroid; UV, ultraviolet.

^a^ Some participants were counted in multiple categories.

^b^ TCS rank classification differs in Japan from that in Europe or the USA, as Japan generally classifies TCS into five ranks: strongest (e.g., 0.05% clobetasol propionate), very strong (e.g., 0.1% mometasone furoate), strong (e.g., 0.3% deprodone propionate), medium (e.g., 0.3% prednisolone valerate acetate), and weak (e.g., 0.5% prednisolone) [3].

^c^ No biologics were used for these participants before commercialization of dupilumab in Japan (in the second year of this study).

^d^ Anti‐allergic drugs included chemical mediator release inhibitors (sodium cromoglicate and tranilast) and a cytokine inhibitor (suplatast tosilate) [25].

The use of systemic anti‐inflammatory agents at baseline was apparently higher among participants with AD‐Pn (any oral agent 18.5%, oral corticosteroids 7.4%, and oral immunosuppressants, i.e., mostly ciclosporin 14.8%) than in those without Pn (10.2%, 2.3%, and 8.5%, respectively; Table 2). A high proportion of participants in both cohorts used antihistamines and/or anti‐allergic drugs (80.6% vs. 82.5%), while the use of ultraviolet phototherapy was similarly low in both cohorts (5.6% in each).

### 3.3 AD‐Pn at Baseline

Among the 108 participants with AD‐Pn, 24.1% had < 10 nodules, 58.3% had ≥ 10 to < 100 nodules, and 17.6% had ≥ 100 nodules (Table 3). More participants with severe AD had ≥ 100 nodules (IGA = 4; 30.2%) than those with moderate AD (IGA = 3; 9.2%); in contrast, fewer participants with severe AD had < 10 nodules (14.0%) than those with moderate AD (30.8%).

**TABLE 3:** Number and size of atopic dermatitis‐associated prurigo nodules at baseline by severity of atopic dermatitis.

	With AD‐Pn at baseline (*n* = 108)	Moderate AD (IGA = 3) (*n* = 65)	Severe AD (IGA = 4) (*n* = 43)
No. of nodules, *n* (%)			
< 10	26 (24.1)	20 (30.8)	6 (14.0)
≥ 10 to < 100	63 (58.3)	39 (60.0)	24 (55.8)
≥ 100	19 (17.6)	6 (9.2)	13 (30.2)
Diameter of the most significant nodule, *n* (%)			
< 2 mm	9 (8.3)	8 (12.3)	1 (2.3)
≥ 2 to < 4 mm	49 (45.3)	35 (53.8)	14 (32.6)
≥ 4 to < 8 mm	27 (25.0)	15 (23.1)	12 (27.9)
≥ 8 mm	23 (21.3)	7 (10.8)	16 (37.2)

Abbreviations: AD, atopic dermatitis; AD‐Pn, atopic dermatitis‐associated prurigo nodules; IGA, Investigator's Global Assessment.

Pn size was also related to AD severity. For example, the most significant Pn was ≥ 8 mm in diameter (i.e., considered as large) in 16 of 43 participants (37.2%) with severe AD versus 7 of 65 participants (10.8%) with moderate AD (Table 3). In contrast, the most significant Pn was < 4 mm in diameter (i.e., considered as small) in 15 of 43 participants (34.9%) with severe AD (IGA = 4) compared with 43 of 65 participants (66.2%) with moderate AD (IGA = 3).

While the baseline weekly average PP‐NRS scores were not affected by the number of Pn in either males or females, these scores tended to increase with increasing Pn size among males, whereas no such clear tendency was observed among females (Figure 1B,C).

### 3.4 Two‐Year Outcomes of AD‐Pn

**FIGURE 2:**
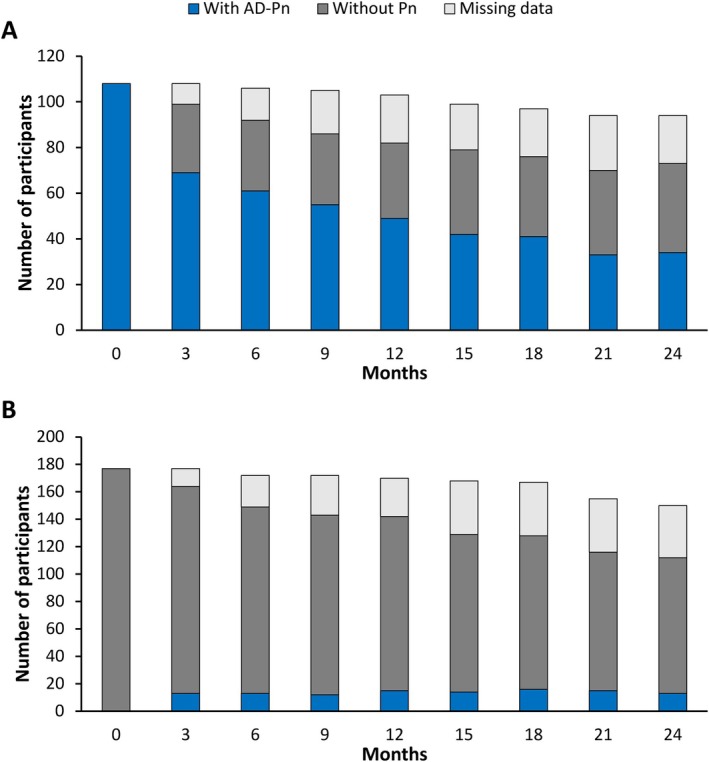
Change in prurigo nodule status from baseline over 2 years in participants (A) with atopic dermatitis‐associated prurigo nodules and (B) without prurigo nodules at baseline. Missing data applied to participants who had not dropped out. AD‐Pn, atopic dermatitis‐associated prurigo nodules; Pn, prurigo nodules.

When the 2‐year outcomes of Pn were assessed, the proportion of participants with AD‐Pn gradually decreased over 2 years (Figure 2A), while the majority of participants without Pn had rarely developed *de novo* Pn during the observation period (Figure 2B). In a subgroup analysis by sex, AD‐Pn was more likely to disappear in males than in females (Figure S2).

### 3.5 Two‐Year Outcomes of AD

**FIGURE 3:**
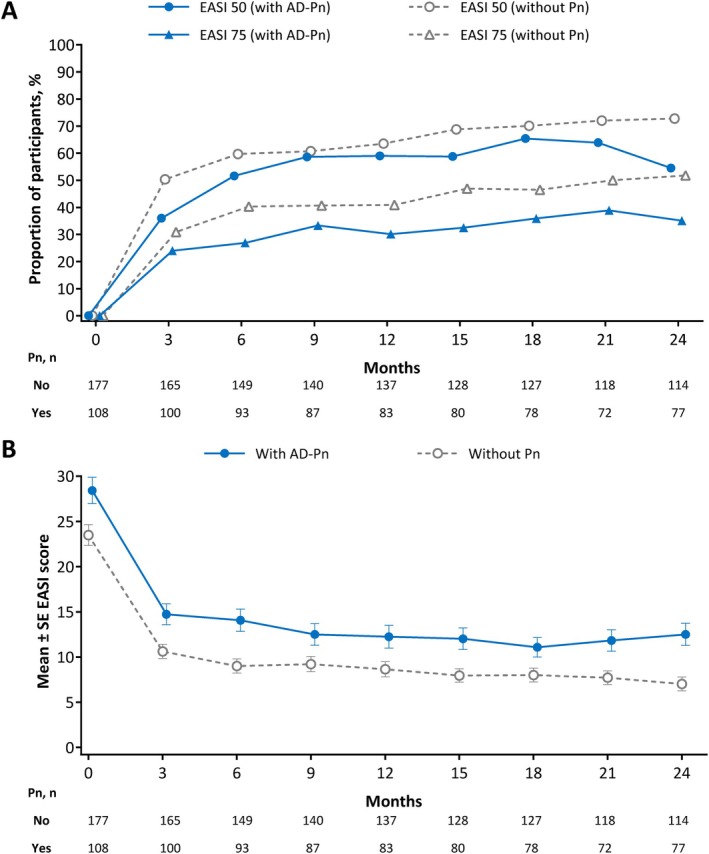
Longitudinal outcomes of AD over 24 months in participants with and without atopic dermatitis‐associated prurigo nodules at baseline for (A) the proportion of participants with ≥ 50% or ≥ 75% improvement in Eczema Area and Severity Index (EASI), and (B) EASI total score. AD‐Pn, atopic dermatitis‐associated prurigo nodules; EASI 50, the proportion of participants with ≥ 50% improvement in EASI; EASI 75, the proportion of participants with ≥ 75% improvement in EASI; Pn, prurigo nodules; SE, standard error.

**FIGURE 4:**
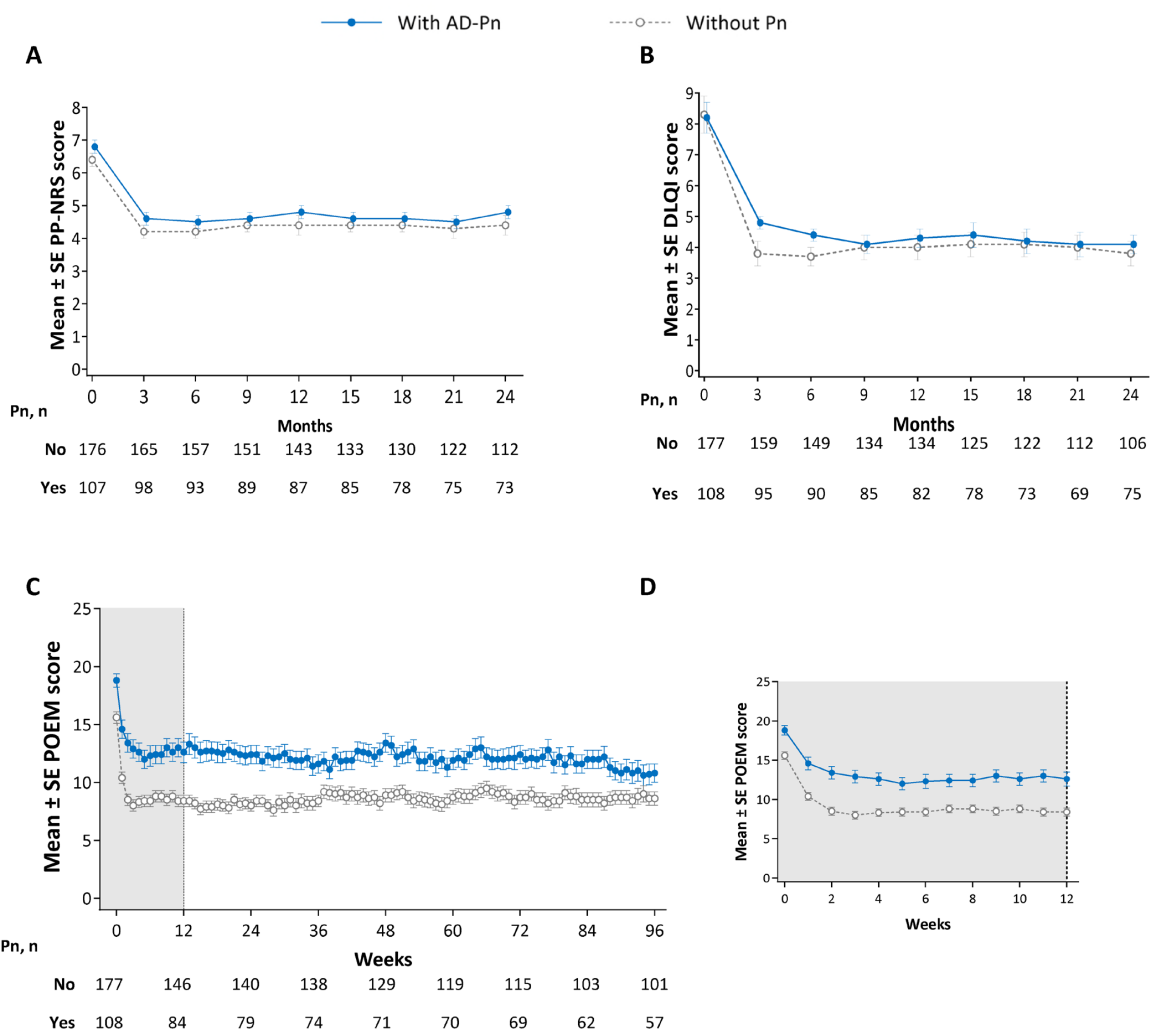
Longitudinal outcomes of peak pruritus NRS score over 24 months in participants with and without atopic dermatitis‐associated prurigo nodules at baseline for (A) average Peak Pruritus Numerical Rating Scale† score, (B) Dermatitis Life Quality Index score, (C) Patient‐Oriented Eczema Measure (POEM) score, and (D) Detailed POEM scores for the first 12 weeks. †Participants were asked for their average PP‐NRS score in the previous 7 days. AD‐Pn, atopic dermatitis‐associated prurigo nodules; DLQI, Dermatology Life Quality Index; Pn, prurigo nodules; POEM, Patient‐Oriented Eczema Measure; PP‐NRS, Peak Pruritus Numerical Rating Scale; SE, standard error.

During 2 years' follow‐up, the proportion of participants who achieved a mean change from baseline in EASI score of ≥ 50% (EASI‐50) or ≥ 75% (EASI‐75) was consistently lower in the cohort with AD‐Pn versus without Pn (Figure 3A). After 2 years, participants with AD‐Pn tended to have more severe AD than participants without Pn, with a mean ± SD percent BSA affected of 31.0% ± 22.5% and 17.9% ± 16.7% for the respective cohorts (Table S2). This lower improvement in participants with AD‐Pn seemed to be attributed to their higher baseline EASI scores, specifically in those registered in dermatology clinics (Figure 3B, Table 1). The changes in weekly mean PP‐NRS scores and DLQI scores were similar over time, regardless of the presence or absence of Pn with consistently higher scores in those with AD‐Pn, with a slight delay in decrease of DLQI score (Month 6) in those with AD‐Pn (Figure 4A,B). These findings were further exemplified by the analysis of EQ‐5D subdomains, where the longitudinal changes in percentages of respondents with any problems in domains of pain/discomfort and anxiety/depression showed consistently smaller improvements in participants with AD‐Pn, indicating these burdens may persist for a longer time in the presence of baseline AD‐Pn (Figure S3). Differences in other subdomains of mobility, self‐care, and usual activities were minimal between groups with and without AD‐Pn at baseline. Repeated collection of weekly POEM scores for 2 years [19] showed a rapid decrease in scores (within 2 weeks) and a subsequent plateau in participants without Pn, whereas participants with AD‐Pn had a more gradual decrease in POEM scores, which plateaued by 5 weeks. Weekly POEM scores were consistently higher over 96 weeks in participants with AD‐Pn than in those without Pn at baseline (Figure 4C,D).

### 3.6 Biomarkers

**FIGURE 5:**
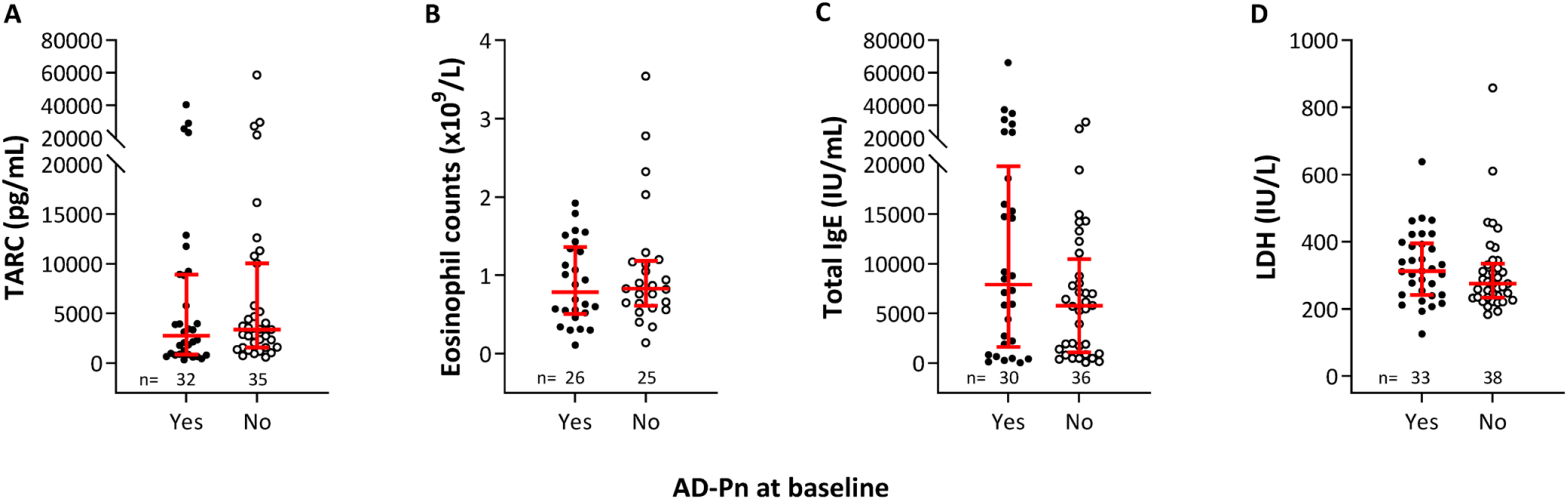
Baseline biomarker levels (in available participants only) for (A) serum thymus‐ and activation‐regulated chemokine levels, (B) peripheral blood eosinophil counts, (C) total immunoglobulin E levels, and (D) serum lactate dehydrogenase levels. The longest red transverse bars indicate median, and error bars indicate interquartile range. AD‐Pn, atopic dermatitis‐associated prurigo nodules; IgE, immunoglobulin E; LDH, lactate dehydrogenase; TARC, thymus‐ and activation‐regulated chemokine.

At baseline, the number of participants with available data for serum TARC, peripheral blood eosinophil counts, serum total IgE, and serum LDH was 67, 51, 66, and 71, respectively. There were no apparent differences in baseline levels of these biomarkers according to the presence or absence of Pn. In participants with AD‐Pn versus those without Pn, median TARC levels were 2755.5 versus 3378.0 pg/mL (Figure 5A), peripheral blood eosinophil counts were 0.784 × 10^9^ versus 0.828 × 10^9^/L (Figure 5B), serum total IgE levels were 7895.0 versus 5770.0 IU/mL (Figure 5C), and serum LDH levels were 313.0 versus 275.0 IU/mL (Figure 5D).

Overall, similar changes in biomarkers were observed over 2 years in participants with AD‐Pn and those without Pn. Serum TARC, eosinophil counts, and serum LDH levels decreased from baseline to 3 months and remained stable during the rest of the study period, whereas total IgE levels fluctuated over time [19]. Serum LDH levels in participants with AD‐Pn tended to be higher than in those without Pn, particularly from 1 year onwards (Figure S4).

### 3.7 Proposed Severity Classification of AD‐Pn

**FIGURE 6:**
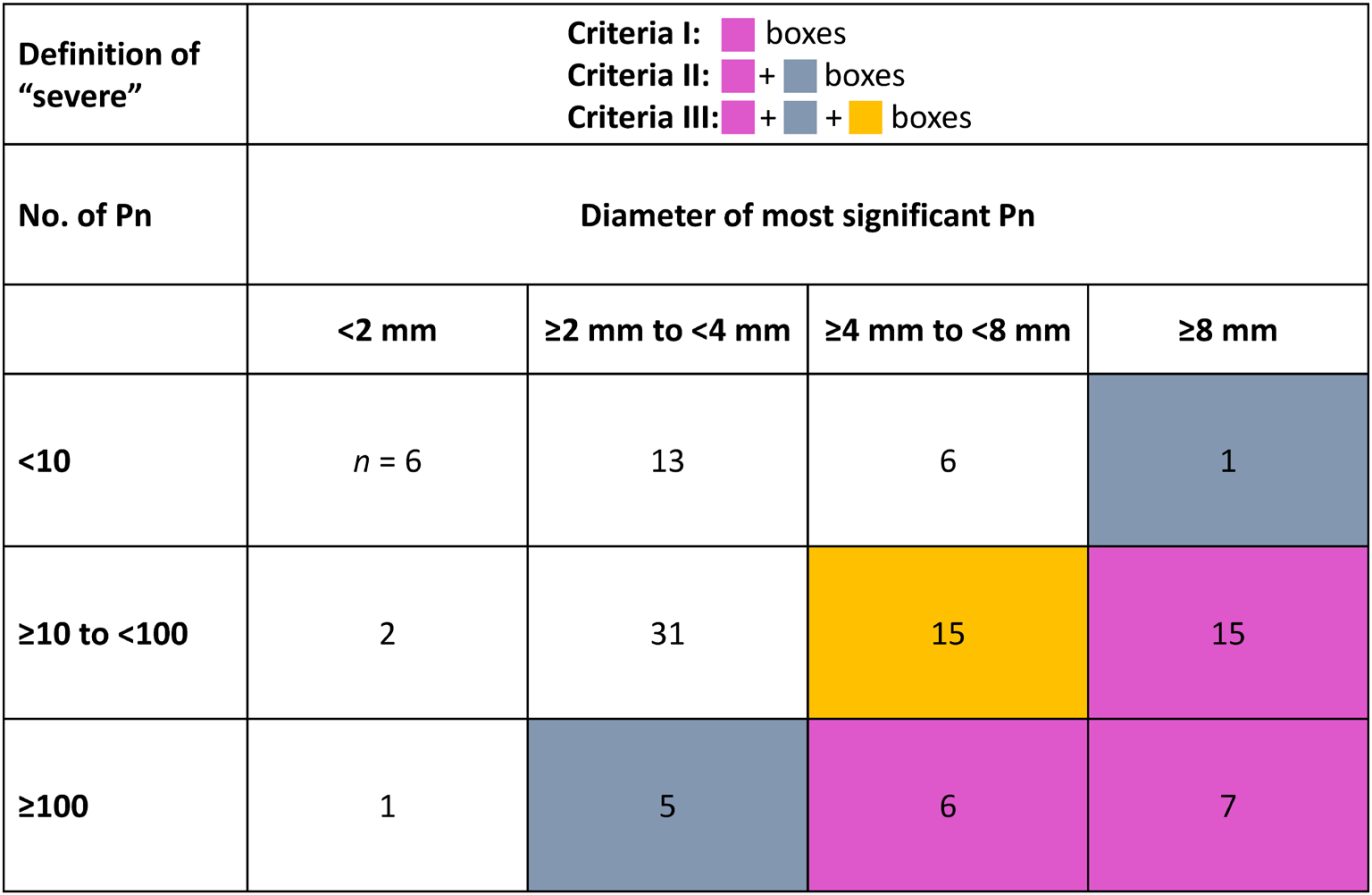
Proposed thresholds for the three severity criteria for “severe” and “non‐severe” prurigo nodules (Pn), and distribution of participants based on size and number of prurigo nodules.

Because there has been no assessment tool developed to determine the severity of AD‐Pn, we propose using collected data. The 108 participants with AD‐Pn were tentatively classified as having “severe” disease by three types of criteria (according to size and number of nodules) to be tested: Criteria I (most stringent): ≥ 100 nodules with the most significant being ≥ 4 mm diameter, or ≥ 10 nodules with the most significant being ≥ 8 mm diameter (*n =* 28); Criteria II: ≥ 100 nodules with the most significant being ≥ 2 mm diameter, or the most significant being ≥ 8 mm diameter regardless of number of nodules (*n =* 34); and Criteria III (least stringent): ≥ 100 nodules with the most significant being ≥ 2 mm diameter, or ≥ 10 nodules with the most significant being ≥ 4 mm diameter, or the most significant being ≥ 8 mm diameter regardless of number of nodules (*n =* 49). For each set of criteria, the remaining participants with AD‐Pn were classified as having “non‐severe” disease (80, 74, and 59 participants, respectively; Figure 6).

**FIGURE 7:**
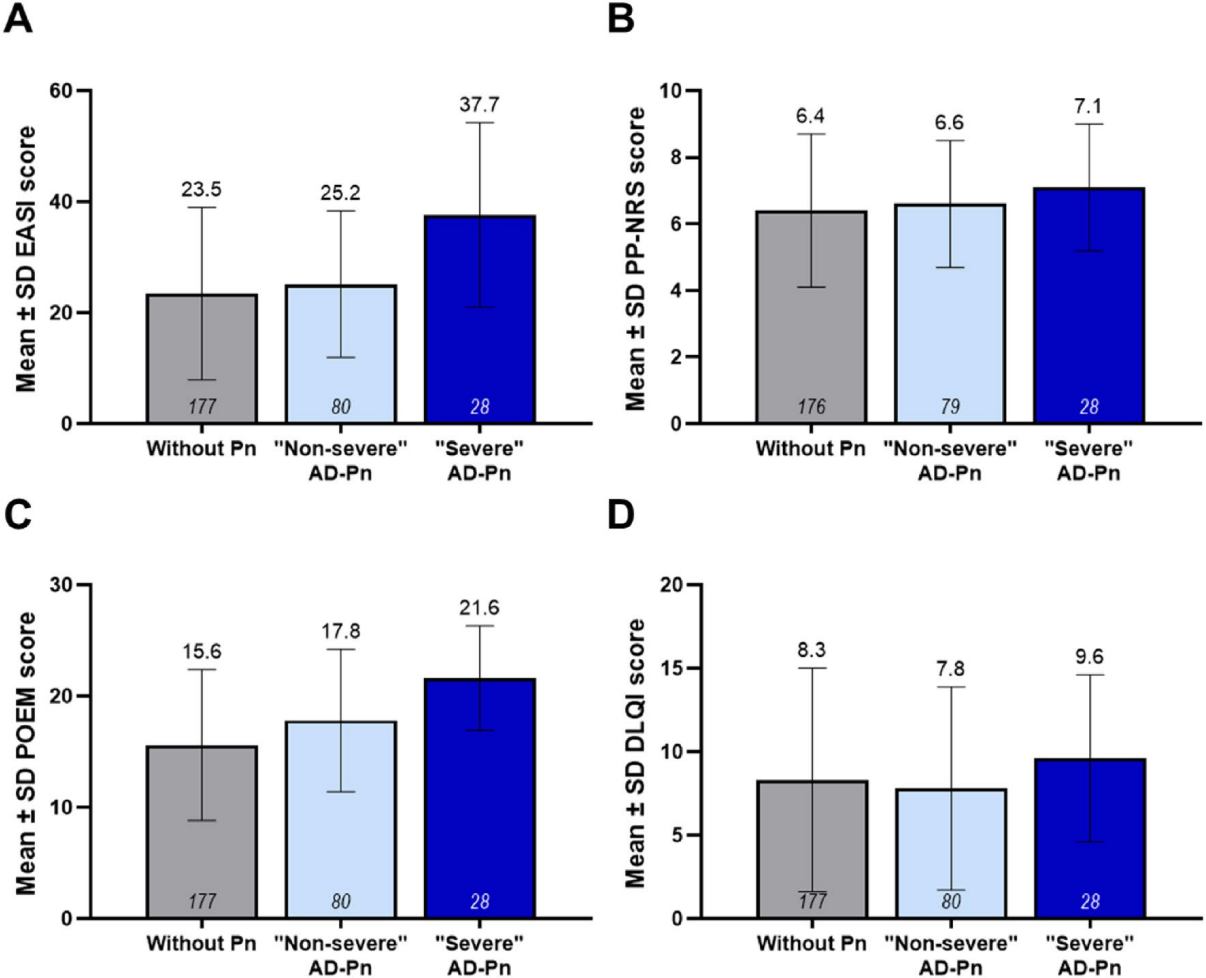
Baseline clinical outcome measure scores by baseline status of prurigo nodules with severity classification by Criteria I for (A) Eczema Area and Severity Index scores, (B) Peak Pruritus Numerical Rating Scale† scores, (C) Patient‐Oriented Eczema Measure scores, and (D) Dermatitis Life Quality Index scores. †Participants were asked for their average PP‐NRS score in the previous 7 days. Italicized numbers indicate sample sizes for each group. Mean values are plotted above the error bars. AD, atopic dermatitis; AD‐Pn, atopic dermatitis‐associated prurigo nodules; DLQI, Dermatitis Life Quality Index; EASI, Eczema Area and Severity Index; Pn, prurigo nodules; POEM, Patient‐Oriented Eczema Measure; PP‐NRS, Peak Pruritus Numerical Rating Scale; SD, standard deviation.

**FIGURE 8:**
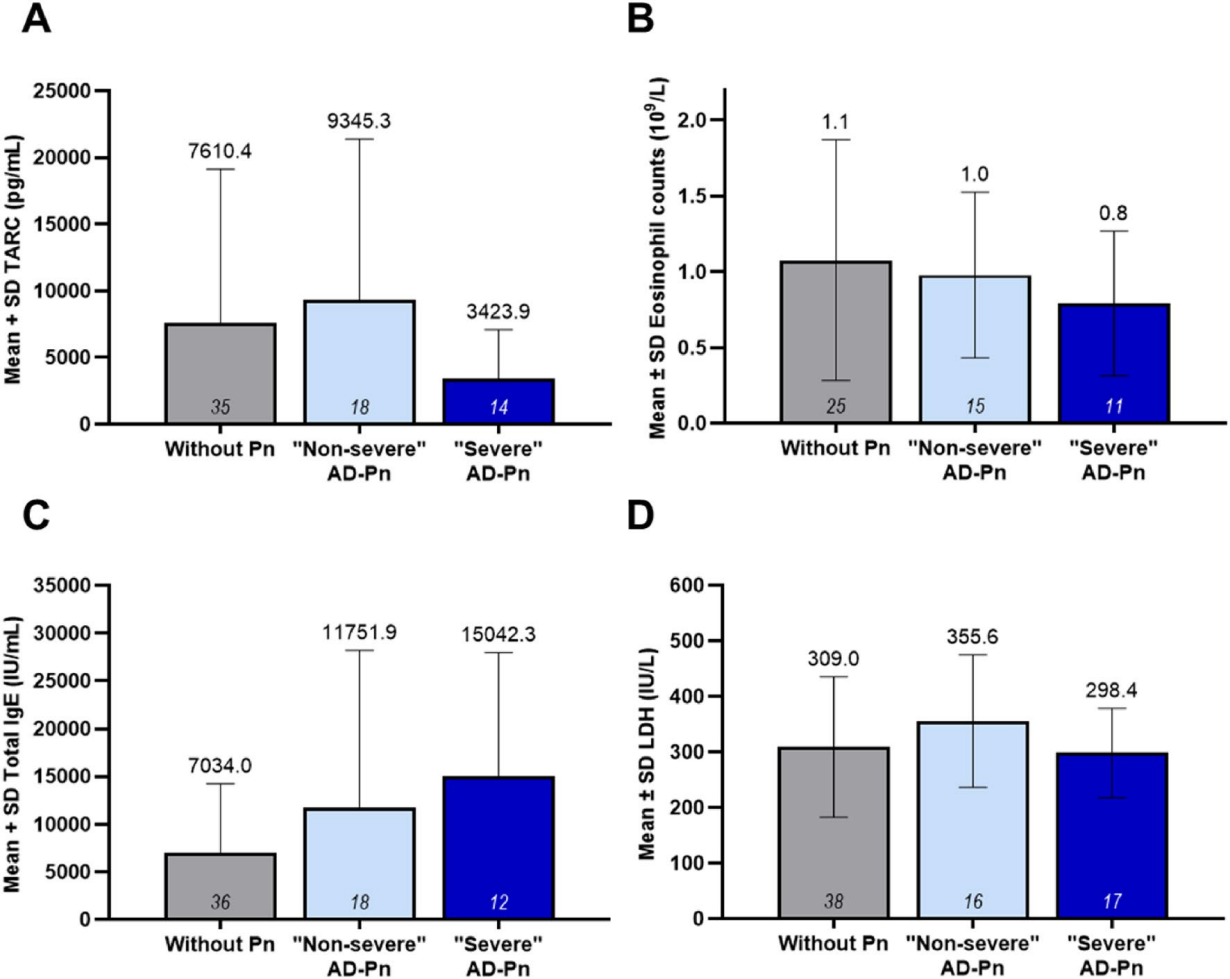
Baseline biomarker levels by baseline status of prurigo nodules with severity classification by Criteria I for (A) serum thymus‐ and activation‐regulated chemokine levels, (B) peripheral blood eosinophils, (C) total immunoglobulin E levels, and (D) serum lactate dehydrogenase levels. Italicized numbers indicate sample sizes for each group. Mean values are plotted above the error bars. AD, atopic dermatitis; AD‐Pn, atopic dermatitis‐associated prurigo nodules; IgE, immunoglobulin E; LDH, lactate dehydrogenase; Pn, prurigo nodules; SD, standard deviation; TARC, thymus‐ and activation‐regulated chemokine.

The total EASI scores (Figure 7A, Figure S5A), PP‐NRS scores (Figure 7B, Figure S5B), POEM scores (Figure 7C, Figure S5C), DLQI scores (Figure 7D, Figure S5D), and biomarker levels (TARC, peripheral blood eosinophils, total IgE, and LDH; Figure 8A–D, Figure S6A–D) were assessed in participants with “severe” and “non‐severe” AD‐Pn according to these three types of criteria and compared with participants without Pn. Although mean baseline scores for EASI, PP‐NRS, POEM, and DLQI were all numerically higher in the “severe” versus “non‐severe” cohort and participants without Pn, the most obvious differences between participants with AD‐Pn and those without Pn were for EASI and POEM scores, irrespective of classification criteria. Baseline serum biomarker levels for TARC, eosinophil counts, and LDH were all numerically lower in the “severe” AD‐Pn versus the “non‐severe” AD‐Pn and no Pn cohorts; total IgE tended to be higher in participants with AD‐Pn, irrespective of the severity of AD‐Pn.

Using Criteria I–III, percent affected BSA tended to be higher for participants in the “severe” versus “non‐severe” cohort, whereas EQ‐5D utility and EQ‐VAS scores were less sensitive for distinguishing between “severe” and “non‐severe” AD‐Pn, and those without Pn (Figure S7).

### 3.8 Treatment and Its Impact on Long‐Term Outcomes of AD by Severity of AD‐Pn

**FIGURE 9:**
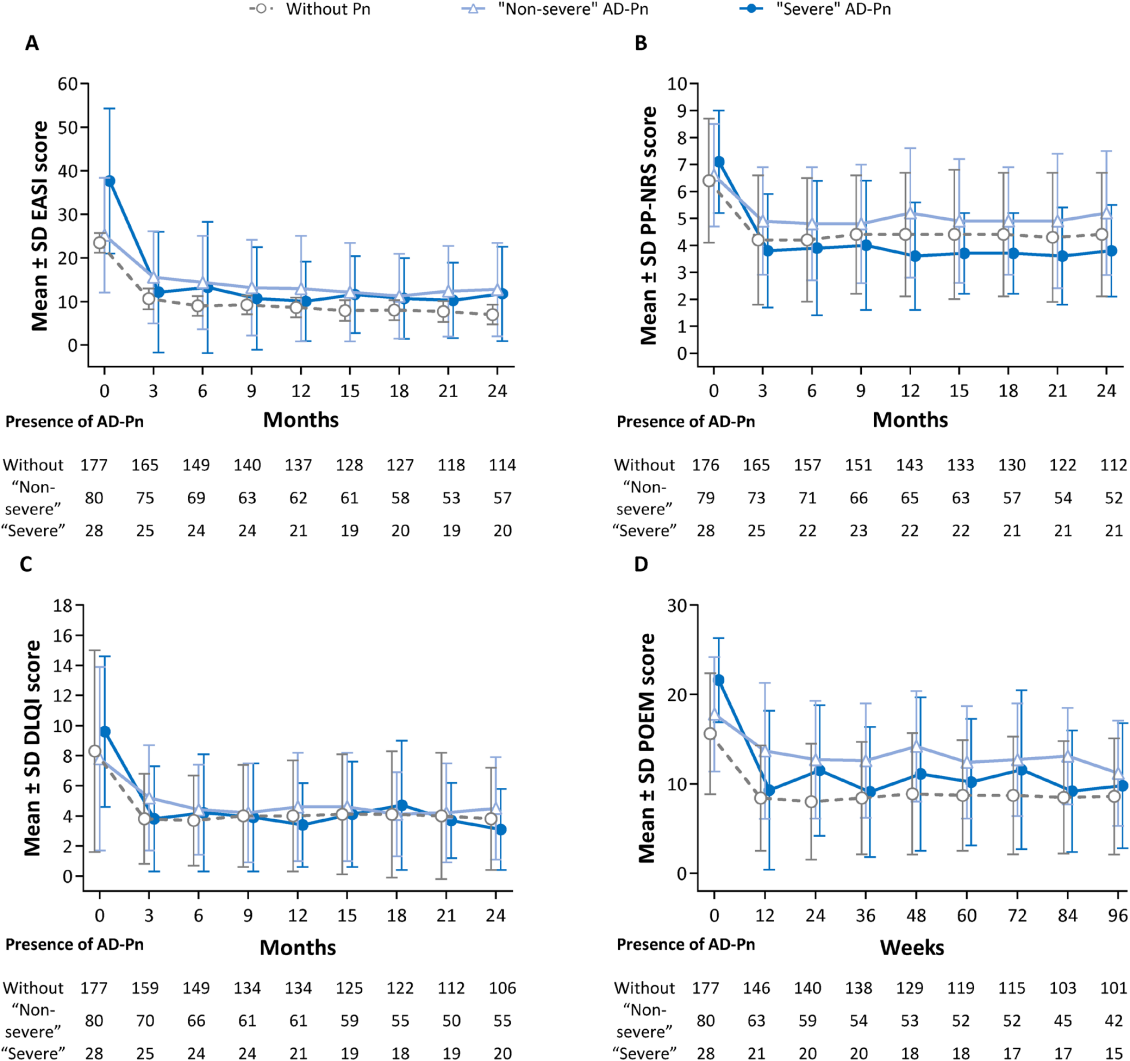
Baseline prurigo nodule status determined long‐term outcomes of atopic dermatitis as assessed by mean ± standard deviation scores for (A) Eczema Area and Severity Index and (B) Peak Pruritus Numerical Rating Scale†, in participants without prurigo nodules and with “non‐severe” and “severe” atopic dermatitis‐associated prurigo nodules according to severity classification Criteria I, and (C) Dermatitis Life Quality Index in participants without prurigo nodules and with “non‐severe” and “severe” atopic dermatitis‐associated prurigo nodules according to severity classification Criteria I over 24 months, and (D) Patient‐Oriented Eczema Measure scores in participants without prurigo nodules and those with “non‐severe” and “severe” atopic dermatitis‐associated prurigo nodules according to severity classification Criteria I over 96 weeks. †Participants were asked for their average PP‐NRS score in the previous 7 days. AD‐Pn, atopic dermatitis‐associated prurigo nodules; DLQI, Dermatitis Life Quality Index; EASI, Eczema Area and Severity Index; POEM, Patient‐Oriented Eczema Measure; PP‐NRS, Peak Pruritus Numerical Rating Scale; Pn, prurigo nodules; SD, standard deviation.

The presence of AD‐Pn was associated with smaller improvement in EASI (Figure 3A,B), while participants with “severe” and “non‐severe” AD‐Pn reached similar levels of EASI scores over 2 years (Figure 9A, Figure S8A). Remarkably, participants with “severe” AD‐Pn showed better improvement than those without Pn in all three criteria for pruritus NRS score (Figure 9B, Figure S8B). Participants with “non‐severe” AD‐Pn had delayed improvement in DLQI by Month 3; however, DLQI scores were eventually similar in participants without Pn and in those with “severe” or “non‐severe” AD‐Pn (Figure 9C, Figure S9A). POEM scores showed a similar trend to PP‐NRS score when Criteria I and II were applied (Figure 9D, Figure S9B).

**FIGURE 10:**
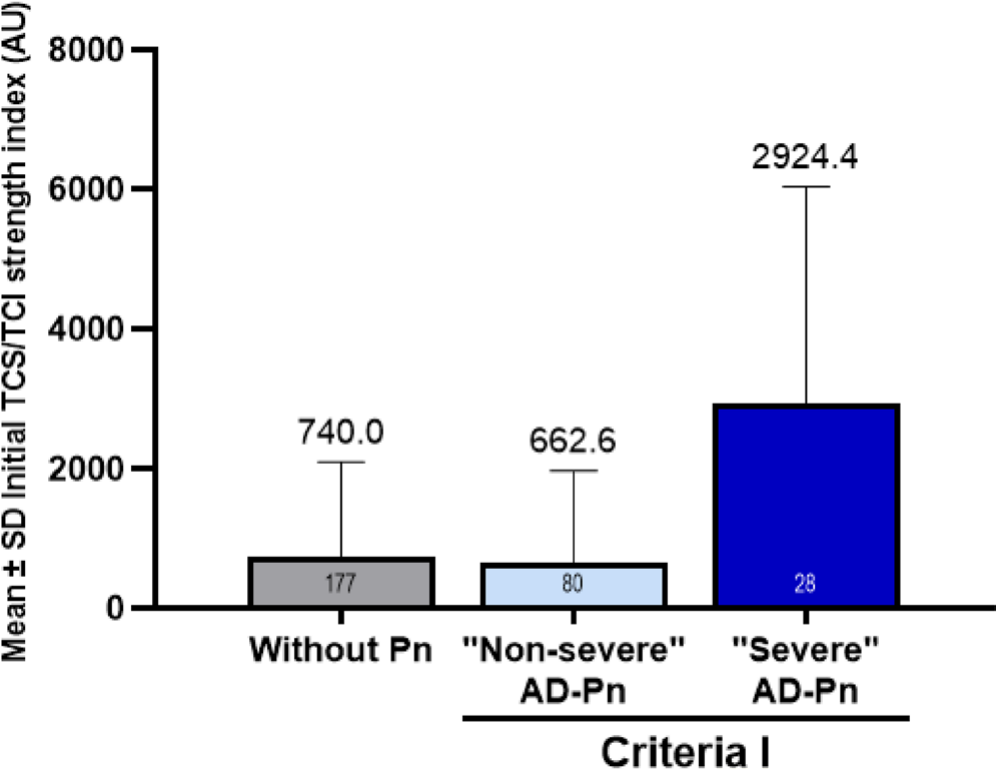
Initial topical corticosteroid/topical calcineurin inhibitor strength index in participants without prurigo nodules and with “non‐severe” and “severe” atopic dermatitis‐associated prurigo nodules according to severity classification Criteria I. Italicized numbers indicate sample sizes for each group. Mean values are plotted above the error bars. AD‐Pn, atopic dermatitis‐associated prurigo nodules; AU, arbitrary units; Pn, prurigo nodules; SD, standard deviation; TCI, topical calcineurin inhibitor; TCS, topical corticosteroid.

To discern the effect of differential treatment on the long‐term outcome between cohorts, initial topical treatment intensity was compared. Participants with “severe” AD‐Pn received more intensive topical TCS/TCI treatment as initial therapy, with a mean ± SD initial TCS/TCI strength index of 2924.4 ± 3114.6 AU (using Criteria I), which was approximately four‐fold higher than that for participants with “non‐severe” AD‐Pn (662.6 ± 1304.2 AU; Figure 10). Similar trends were seen in the “severe” and “non‐severe” AD‐Pn cohorts when Criteria II and III were used for classification (Figure S10). Participants without Pn received a similar intensity of topical TCS/TCI treatment (mean ± SD 740.0 ± 1359.4 AU) as those with “non‐severe” AD‐Pn.

Participants with “severe” AD‐Pn were more often treated in hospitals (with ≥ 20 administration beds) than in dermatology clinics: the proportions of participants with “severe” AD‐Pn in Criteria I, II, and III who were registered in hospitals were 78.6%, 76.5%, and 73.5%, respectively. Overall, participants with AD‐Pn were registered in hospitals (54.6%) and dermatology clinics (45.4%; Table 4).

**TABLE 4:** Number of participants registered across different care providers by severity type.

Institution	With AD‐Pn at baseline (*n* = 108)	“Severe” AD‐Pn by different criteria^a^		
		Criteria I (*n* = 28)	Criteria II (*n* = 34)	Criteria III (*n* = 49)
Hospital^b^, *n* (%)	59 (54.6)	22 (78.6)	26 (76.5)	36 (73.5)
Dermatology clinic, *n* (%)	49 (45.4)	6 (21.4)	8 (23.5)	13 (26.5)

Abbreviation: AD‐Pn, atopic dermatitis‐associated prurigo nodules.

^a^ Participants with AD‐Pn were tentatively classified as having “severe” disease by three types of proposed criteria (according to size and number of nodules): Criteria I (most stringent): ≥ 100 prurigo nodules with the most significant being ≥ 4 mm diameter, or ≥ 10 nodules with the most significant being ≥ 8 mm diameter; Criteria II: ≥ 100 nodules with the most significant being ≥ 2 mm diameter, or the most significant being ≥ 8 mm diameter regardless of the number of nodules; and Criteria III (least stringent): ≥ 100 nodules with the most significant being ≥ 2 mm diameter, or ≥ 10 nodules with the most significant being ≥ 4 mm diameter, or the most significant being ≥ 8 mm diameter regardless of the number of nodules.

^b^ A hospital was defined as a medical institution with ≥ 20 administration beds.

## 4 Discussion

We report the baseline characteristics of individuals with AD‐Pn and their progression in signs and symptoms over 2 years in the ADDRESS‐J registry of Japanese adults with moderate‐to‐severe AD. By exploring various aspects, we sought to reveal what characterizes AD‐Pn, and the clinical relevance of recognizing AD‐Pn.

The current study findings indicated that the impact of the number of nodules varied among participants in terms of degree of pruritus by PP‐NRS scores, suggesting that the number of nodules alone may not be a good indication of severity in patients with AD‐Pn. This is in sharp contrast to the established severity measures in PN, where the number of nodules is regarded as a good surrogate marker for disease severity (e.g., IGA PN‐Stage) [16, 26]. Instead, baseline AD may contribute to a significant portion of the pruritus experienced by individuals with AD‐Pn.

In reality, participants with AD‐Pn had a higher baseline use of the strongest‐potency TCS and oral corticosteroids or oral immunosuppressants (mostly ciclosporin) than those without Pn, suggesting AD‐Pn itself is an indicator of refractory AD. Ultraviolet phototherapy is recommended for use in treating participants with AD uncontrolled with topical therapy in the treatment guidelines, but the proportions of participants using ultraviolet phototherapy at baseline did not differ between the two cohorts in the present results (5.6% in each). This low rate may reflect the lack of sufficient study locations, as phototherapy requires a special medical device and frequent clinic visits to the practitioners.

Pn arise from AD lesions and may affect the treatment course of AD [1]. Thus, we looked at 2‐year outcomes of AD by the presence or absence of Pn at baseline. The presence of AD‐Pn was consistently associated with poorer outcomes over 2 years, as measured by EASI and POEM scores. This was explained in part by the higher baseline scores compared with participants without Pn. In contrast, scores for pruritus and skin‐associated QoL were not greatly affected by the presence of AD‐Pn. The high POEM score, which assesses eczema symptoms from the individual's perspective, reveals that symptom perception was apparent, but DLQI and EQ‐5D scores were low. The obvious lack of relationship between AD‐Pn and those QoL outcomes may be explained by the fact that these burdens were mainly driven by AD. In other words, the presence of moderate‐to‐severe AD has a more dominant role than AD‐Pn per se in contributing to the symptomatic and mental burden of disease at a certain point. Nevertheless, it has been reported that skin diseases accompanied by pruritus such as PN are more likely to be associated with mental comorbidities than skin diseases without pruritus [27, 28].

In the present analysis, although participants with AD‐Pn showed persistently higher proportions of reported problems in the EQ‐5D anxiety/depression domain over the 2‐year follow‐up, these findings should be interpreted cautiously. As formal psychiatric diagnoses and psychotropic medication use were not assessed, the results reflect self‐reported emotional distress captured by the EQ‐5D rather than confirmed psychiatric comorbidity. Accordingly, the observed differences suggest a greater perceived psychological burden associated with AD‐Pn, but do not allow inference regarding the presence or persistence of clinically diagnosed psychiatric disorders.

In this 2‐year study in participants with moderate‐to‐severe AD, very few new nodules developed in those without Pn (approximately 10% of participants). This indicates that it may take years for Pn to develop in people with AD, or that those without Pn were not intrinsically susceptible to suffering from AD‐Pn. Our study observed that in participants with AD‐Pn, these nodules gradually disappeared, with approximately half of this cohort having no nodules by 2 years (end of study observation). Importantly, AD had improved from baseline after approximately 3 months, whereas the clearance of Pn took more time. This is in line with the notion that the pathophysiology of AD‐Pn is distinct from that of AD [1].

Among participants with AD‐Pn, we tried to discern those with “severe” AD‐Pn from those with “non‐severe” AD‐Pn. Such criteria are needed, with the emergence of novel treatment modalities for severe AD. Based on data from the present AD‐Pn cohort, we propose a tentative severity classification for AD‐Pn, which may serve as a framework for the assessment of treatment effectiveness in this setting.

Apparent paradoxical responses by AD‐Pn severity were observed for EASI, PP‐NRS, and POEM scores (except with Criteria III), where “severe” AD‐Pn showed better treatment outcomes than “non‐severe” AD‐Pn. This was partly explained by the difference in treatment intensity. In the present analysis, participants with “severe” AD‐Pn in any criteria group received ≥ 4‐fold more intensive initial topical treatments, which corresponded to higher treatment effectiveness in AD, as measured by EASI, PP‐NRS, and POEM scores. As stated earlier, QoL measures are affected predominantly by AD and having AD‐Pn seems to add only marginal QoL‐associated burden to the participants when measured by DLQI, EQ‐5D, and EQ‐VAS scores at baseline. At the beginning of this study, there was no advanced targeted therapy available for people with AD beyond ciclosporin in Japan, and the main treatment modality was topical medicines, regardless of Pn severity. How the results of the present analyses on target treatment affect the management landscape of AD‐Pn will be a topic for future study.

Of note, individuals with more severe AD‐Pn more frequently receive treatment in hospitals and, therefore, the participants in this study may have received more intensive treatment than those treated in dermatology clinics. In the entire analysis set of ADDRESS‐J, 46.7% of participants were registered in hospitals and the remainder (53.3%) were in dermatology clinics [2]. Among those with AD‐Pn, the proportion of participants in hospital or dermatology clinic populations was slightly weighted in favor of those registered in hospitals, and more so among those with “severe” AD‐Pn based on our tentative severity classifications. This indicates that participants with “severe” AD‐Pn were biased toward hospital‐based treatment that differs from that received by participants registered in dermatology clinics. When considering the three possible severity criteria, it was difficult to choose one set of criteria over the others due to the limited numbers of participants. Nevertheless, we propose starting with the most stringent criteria (Criteria I) to assess AD‐Pn, as those with a diameter of < 4 mm may be less significant than larger nodules, and there may be very few individuals who have < 10 nodules that are > 8 mm in diameter.

It is important to state the distinction between AD‐Pn and PN. Based on the present findings, “severe” AD‐Pn is associated with higher baseline EASI and POEM scores and greater percent affected BSA compared to “non‐severe” AD‐Pn. On the contrary, the skin lesions in PN exist alone and have little to contribute to the severity of the EASI score and BSA involvement, suggesting that AD‐Pn is a part of the surrounding dermatitis and occurs during the process of highly active AD inflammation. From an epidemiological aspect, PN has been reported to be more prevalent in females [29], whereas, in our cohort, AD‐Pn appears more prevalent in males. These differences further support that AD‐Pn and PN have distinct backgrounds. Interestingly, baseline PP‐NRS scores indicated that males experienced more pruritus as the size of the nodule increased, whereas this tendency was not observed in female participants. Further study in larger population samples will be needed to confirm this sex‐specific pruritus sensitivity in AD‐Pn.

Whether any relationships exist between disease measures and severity of AD‐Pn is also an outstanding question to address. Participants with “severe” AD‐Pn had lower TARC levels, eosinophil counts, and LDH levels at baseline than those with “non‐severe” AD‐Pn, which may reflect more intensive background medical care in the former population; however, this is yet to be confirmed due to the low numbers of participants with evaluable measurements from each population. Irrespective of “severity”, serum total IgE levels tended to be higher in participants with AD‐Pn than in those without Pn, which may correspond to the refractory nature of AD‐Pn to conventional topical treatments. However, it also remains unclear whether elevated IgE levels are involved in the pathogenesis of AD‐Pn, particularly since very few participants had evaluable baseline measurements in each cohort. Lastly, the POEM score appears to be the most sensitive for distinguishing between those with “severe” and “non‐severe” AD‐Pn, as participants with “non‐severe” AD‐Pn showed the poorest 2‐year outcomes compared with those with “severe” AD‐Pn. This may mean that participants with “non‐severe” AD‐Pn received suboptimal treatment because of their seemingly milder clinical appearance. Recognition and intensive management of AD‐Pn irrespective of its severity is crucial to achieve better long‐term disease control of AD.

The main limitations of this registry study were the number of discontinuations and missing data during the 2‐year study period and the relatively small number of participants with AD‐Pn. Baseline characteristics for the 239 participants who remained in the study through the observation period were generally similar to those of the overall population (Table S3). Nevertheless, because reasons for discontinuation were not fully captured and residual confounding cannot be ruled out, the possibility of attrition bias cannot be excluded. Additionally, the availability of biomarker data was limited. The *post hoc* design and descriptive approach preclude any causal inference and may limit the generalizability of the findings. It is also important to note that the enrolled participants had moderate‐to‐severe disease at baseline, which may underrepresent the typical real‐world AD population with milder disease. These limitations need to be considered when interpreting the findings of this study.

In conclusion, AD‐Pn was found to be more prevalent in males in the Japanese ADDRESS‐J registry cohort and served as an indicator of treatment‐refractory AD. Participants with AD‐Pn showed poor therapeutic response to conventional (primarily topical) treatments in AD and experienced suboptimal long‐term AD prognosis over 2 years even after receiving intensive initial therapy. While there might have been inappropriate management of participants with “non‐severe” AD‐Pn, the present findings suggest that AD‐Pn could serve as a valuable indicator for transitioning from topical to more advanced systemic therapy to achieve better long‐term AD management. Furthermore, although limited in sample size, participants with “severe” AD‐Pn receiving intensive topical treatment showed reductions in TARC, LDH, and peripheral eosinophil counts, but notably, total IgE levels remained elevated. This observation warrants further investigation into the potential role of persistently elevated total IgE levels in the chronicity of AD‐Pn. Additionally, we propose a tentative severity classification of AD‐Pn based on the number and size of the nodules and the PROs from the ADDRESS‐J registry cohort, which may serve as a framework for further evaluation of treatment response of AD‐Pn.

## Funding

This research was funded and sponsored by Sanofi K.K. Development of the manuscript and the open access publishing fee were supported by Sanofi and Regeneron. The primary study was conducted in collaboration with the Steering Committee, which included Norito Katoh, Hidehisa Saeki, Takafumi Etoh, Yoko Kataoka, and Satoshi Teramukai. While the Steering Committee meetings were funded by Sanofi K.K., the authors were not paid for the publication process.

## Ethics Statement

Approval of the research protocol by Institutional Reviewer Boards/Ethics Committees: Asai Dermatology Clinic Institutional Review Board (Central IRB for dermatology clinics, DIREGL07886); Kyoto Prefectural University of Medicine Medical Ethics Review Committee (ERB‐C‐668); Nippon Medical School Ethics Committee (28–12‐685); Medical Research Ethics Committee/Commissioned Research Review Committee, Osaka Habikino Medical Center (FuByouKo160‐07_02); Medical Ethics Committee, Tokyo Teishin Hospital (Rin‐1001); Epidemiological Research Ethics Review Committee, Graduate School of Biomedical and Health Sciences, Hiroshima University (E‐685); Ethics Committee, Tokyo Women's Medical University (ID n/a); Epidemiology and Observational Research Ethics Review Committee, The University of Tokyo Graduate School of Medicine (11418); Intervention and Observational Research Ethics Review Committee, Osaka University Graduate School of Medicine (ID n/a); University of Yamanashi School of Medicine Ethics Committee (1626); Clinical Research Ethics Review Committee, Dokkyo Medical University Saitama Medical Center (1652).

## Consent

All participants completed written informed consent forms before initiating any study procedure.

## Conflicts of Interest

Yoko Kataoka has received honoraria for lectures from AbbVie, Sanofi, Maruho, and Pfizer; and research grants from Sanofi, AbbVie, Amgen, Eli Lilly Japan, LEO Pharma, Maruho, Otsuka Pharmaceutical, Taiho Pharmaceutical, and Pfizer Japan. Takafumi Etoh has received honoraria for lectures from Kyowa Kirin and Maruho. Hidehisa Saeki has received lecture fees from AbbVie, Amgen, Eli Lilly Japan, Japan Tobacco, LEO Pharma, Maruho, Mitsubishi Tanabe Pharma, Novartis Pharma, Otsuka Pharmaceutical, Pfizer Japan, Sanofi K.K., Taiho Pharmaceutical, Nippon Boehringer Ingelheim, and Torii Pharmaceutical; clinical research funding from LEO Pharma; grant donations from Eisai, Maruho, Taiho Pharmaceutical, Tokiwa Pharmaceutical, and Torii Pharmaceutical. Norito Katoh has received honoraria as a speaker/consultant for Sanofi, Maruho, AbbVie, Eli Lilly Japan, Taiho Pharmaceutical, Pfizer, Mitsubishi Tanabe Pharma, Janssen Pharma, Kyowa Kirin, Celgene Japan, and Otsuka Pharmaceutical; has received grants as an investigator from Mitsubishi Tanabe Pharma, Torii Pharmaceutical, Maruho, Sun Pharma, Boehringer Ingelheim Japan, Eisai, and LEO Pharma. Hidehisa Saeki and Norito Katoh are Editorial Board members of *The Journal of Dermatology* and as such were excluded from all editorial decisions related to the acceptance of this article for publication. Satoshi Teramukai has received honoraria for lectures from Chugai Pharmaceutical, and Nippon Boehringer Ingelheim; research grants from Sun Contact Lens; and consultant fees from Atworking, Daiichi Sankyo, Eye‐lens, Human Life CODE, Kaneka, Kringle Pharma, Sanofi K.K., Sound Wave Innovation, Solasia Pharma, and Sysmex. Yuki Tajima, Kwinten Bosman, and Kazuhiko Arima are Sanofi K.K. employees and may hold stock and/or stock options in the company. Marius Ardeleanu is an employee and shareholder of Regeneron Pharmaceuticals Inc.

Yoko Kataoka, Takafumi Etoh, Hidehisa Saeki, Norito Katoh, Satoshi Teramukai, Yuki Tajima, Marius Ardeleanu, and Kazuhiko Arima contributed to the study concept and design. Yoko Kataoka, Takafumi Etoh, Hidehisa Saeki, and Norito Katoh acquired the data. All authors had full access to the data, contributed to the data analysis or interpretation, and participated in the development, review, approval, and decision to submit this publication; this decision rested with the authors, independently of the sponsor. All authors read and approved the final version. All named authors meet the International Committee of Medical Journal Editors (ICMJE) criteria for authorship for this article, take responsibility for the integrity of the work as a whole, and have given their approval for this version to be published.

## Supporting information


**Table S1:** Summary of baseline mental characteristics based on anxiety/depression domain of EQ‐5D.
**Table S2:** Outcomes at 2 years for participants with atopic dermatitis‐associated prurigo nodules and those without prurigo nodules at baseline.
**Table S3:** Summary of baseline demographic and participant characteristics for participants who remained in the study through the observation period (without formal discontinuation).
**Figure S1:** Participant flow diagram.
**Figure S2:** Numbers of participants with atopic dermatitis‐associated prurigo nodules over time in (A) males and (B) females with atopic dermatitis‐associated prurigo nodules at baseline, and in (C) males and (D) females without prurigo nodules at baseline. Missing data applied to participants who had not dropped out.
**Figure S3:** Longitudinal changes in percentages of respondents with any problems in each EuroQOL group health questionnaire with five dimensions (EQ‐5D) subdomain by baseline presence of atopic dermatitis‐associated prurigo nodules.
**Figure S4:** Change in biomarkers over time by baseline presence of atopic dermatitis‐associated prurigo nodules for (A) serum thymus‐ and activation‐regulated chemokine levels, (B) peripheral blood eosinophil count, (C) total immunoglobulin E levels, and (D) serum lactate dehydrogenase levels.
**Figure S5:** Distribution of participants with atopic dermatitis‐associated prurigo nodules at baseline according to severity classification Criteria II and III for (A) Eczema Area and Severity Index scores, (B) Peak Pruritus Numerical Rating Scale scores, (C) Patient‐Oriented Eczema Measure scores, and (D) Dermatitis Life Quality Index scores.
**Figure S6:** Distribution of participants with atopic dermatitis‐associated prurigo nodules at baseline according to severity classification Criteria II and III for (A) serum thymus‐ and activation‐regulated chemokine levels, (B) peripheral blood eosinophils, (C) total immunoglobulin E levels, and (D) serum lactate dehydrogenase levels.
**Figure S7:** Mean values for (A) percent affected body surface area, (B) EuroQOL questionnaire with 5 dimensions utility score, and (C) EuroQOL questionnaire with visual analog scale score among participants with “non‐severe” and “severe” atopic dermatitis‐associated prurigo nodules and participants without prurigo nodules.
**Figure S8:** Baseline prurigo nodule status determined long‐term outcomes of atopic dermatitis as assessed by mean ± standard deviation scores for (A) Eczema Area and Severity Index, and (B) Peak Pruritus‐Numerical Rating Scale, in participants without prurigo nodules and with “non‐severe” and “severe” atopic dermatitis‐associated prurigo nodules according to severity classification Criteria II and III.
**Figure S9:** Baseline prurigo nodule status determined long‐term outcomes of atopic dermatitis as assessed by mean ± standard deviation scores for (A) Dermatitis Life Quality Index in participants without prurigo nodules and with “non‐severe” and “severe” atopic dermatitis‐associated prurigo nodules according to severity classification Criteria II and III over 24 months, and (B) Patient‐Oriented Eczema Measure scores in participants without prurigo nodules and those with “non‐severe” and “severe” atopic dermatitis‐associated prurigo nodules according to severity classification Criteria II and III over 96 weeks.
**Figure S10:** Initial topical corticosteroid/topical calcineurin inhibitor strength index in participants without prurigo nodules and with “non‐severe” and “severe” atopic dermatitis‐associated prurigo nodules according to severity classification Criteria II and III.

## Data Availability

Qualified researchers may request access to study documents (including the clinical study report, study protocol with any amendments, and statistical analysis plan) that support the methods and findings reported in this manuscript. Individual anonymized participant data will be considered for sharing if there is legal authority to share the data and there is not a reasonable likelihood of participant re‐identification. Submit requests to https://vivli.org/.
