# Supplementary material for: Characterization of Prurigo Nodules in Adults With Moderate‐To‐Severe Atopic Dermatitis in Japan: A 2‐Year Observational Study
Source: J Dermatol. 2026 Jul 2;53(8):1099–114. doi: 10.1111/1346-8138.70345 (PMC13435008; doi:10.1111/1346-8138.70345)
Supplement: Supplementary file 1 [file JDE-53-1099-s001.docx]

**Characterization of prurigo nodules in adults with moderate-to-severe atopic dermatitis in Japan: a 2-year observational study**

**Authors:** Yoko Kataoka^1^, Takafumi Etoh^2,3^, Hidehisa Saeki^4^, Norito Katoh^5^, Satoshi Teramukai^6^, Yuki Tajima^7^, Kwinten Bosman^8^, Marius Ardeleanu^9^, Kazuhiko Arima^7^

**Affiliations:** ^1^Department of Dermatology, Osaka Habikino Medical Center, Osaka, Japan; ^2^Atago Dermatology Clinic, Tokyo, Japan; ^3^Department of Dermatology, Tokyo Teishin Hospital, Tokyo, Japan; ^4^Department of Dermatology, Nippon Medical School, Tokyo, Japan; ^5^North Campus, Kyoto Prefectural University of Medicine, Kyoto, Japan; ^6^Department of Biostatistics, Kyoto Prefectural University of Medicine, Kyoto, Japan; ^7^Sanofi K.K., Tokyo, Japan; ^8^Sanofi, Cambridge, MA, USA; ^9^Regeneron Pharmaceuticals, Inc., Tarrytown, NY, USA

**Running title:** Atopic dermatitis with prurigo nodules

**Corresponding author:** Yoko Kataoka, Department of Dermatology, Osaka Habikino Medical Center, 3-7-1 Habikino, Habikino City, Osaka 583-8588, Japan

**Tel:** +81-72-957-2121; **Email:** kataokay@ra.opho.jp; **ORCID ID:** 0000-0002-5121-0134

## TABLE S1

Summary of baseline mental characteristics based on anxiety/depression domain of EQ-5D.

|  | **AD-Pn at baseline** | | | | | |
| --- | --- | --- | --- | --- | --- | --- |
| **Anxiety/depression, n (%)** | **Yes (n = 108)** | | | **No (n = 177)** | | |
|  | **Overall (n = 108)** | **Hospital  (n = 59)** | **Dermatology clinic (n = 49)** | **Overall (n = 177)** | **Hospital (n = 74)** | **Dermatology clinic (n = 103)** |
| Not anxious/depressed | 58 (53.7) | 25 (42.4) | 33 (67.3) | 102 (57.6) | 24 (32.4) | 78 (75.7) |
| Slightly anxious/depressed | 28 (25.9) | 17 (28.8) | 11 (22.4) | 43 (24.3) | 25 (33.8) | 18 (17.5) |
| Moderately anxious/depressed | 13 (12.0) | 8 (13.6) | 5 (10.2) | 17 (9.6) | 11 (14.9) | 6 (5.8) |
| Severely anxious/depressed | 6 (5.6) | 6 (10.2) | 0 | 9 (5.1) | 8 (10.8) | 1 (1.0) |
| Extremely anxious/depressed | 3 (2.8) | 3 (5.1) | 0 | 5 (2.8) | 5 (6.8) | 0 |
| Missing | 0 | 0 | 0 | 1 (0.6) | 1 (1.4) | 0 |

AD-Pn, atopic dermatitis-associated prurigo nodules; EQ-5D, EuroQOL group health questionnaire with five dimensions.

## TABLE S2

Outcomes at 2 years for participants with atopic dermatitis-associated prurigo nodules and those without prurigo nodules at baseline.

| **Outcome** | **Baseline** | | **At 2 years** | |
| --- | --- | --- | --- | --- |
|  | **AD-Pn at baseline (*n* = 108)** | **Without Pn at baseline (*n* = 177)** | **AD-Pn at baseline (*n* = 93)*** | **Without Pn at baseline (*n* = 146)*** |
| With AD-Pn, *n* (%)† | 108 (100) | 0 | 34 (46.6) | 13 (11.6) |
| IGA score, *n* (%)‡ |  |  |  |  |
| 0 | 0 | 0 | 2 (2.6) | 10 (8.7) |
| 1 | 0 | 0 | 3 (3.9) | 16 (13.9) |
| 2 | 0 | 0 | 29 (37.7) | 51 (44.3) |
| 3 | 65 (60.2) | 144 (81.4) | 36 (46.8) | 37 (32.2) |
| 4 | 43 (39.8) | 33 (18.6) | 7 (9.1) | 1 (0.9) |
| EASI score§ | 28.4 ± 15.1 | 23.5 ± 15.5 | 12.5 ± 10.7 | 7.0 ± 8.1 |
| EASI 50, *n* (%) | – | – | 42 (54.5) | 83 (72.8) |
| EASI 75, *n* (%) | – | – | 27 (35.1) | 59 (51.8) |
| Percent BSA affected, % § | 53.9 ± 23.2 | 49.0 ± 24.5 | 31.0 ± 22.5 | 17.9 ± 16.7 |
| Weekly average PP-NRS score¶† | 6.8 ± 1.9 | 6.4 ± 2.3 | 4.8 ± 2.2 | 4.4 ± 2.3 |
| POEM score†† | 18.8 ± 6.2 | 15.6 ± 6.8 | 10.8 ± 6.2 | 8.6 ± 6.5 |
| DLQI score‡‡ | 8.2 ± 5.8 | 8.3 ± 6.7 | 4.1 ± 3.3 | 3.8 ± 3.4 |

*Note:* Data are presented as mean ± SD unless stated otherwise. Missing data for each score/item were censored at 2 years.
*The given numbers of participants at 2 years were those who did not explicitly discontinue the study.

†AD-Pn and PP-NRS scores were assessed in 73 and 112 participants for groups with and without AD-Pn, respectively, at 2 years.
‡IGA scores were assessed in 77 and 115 participants for groups with and without AD-Pn, respectively, at 2 years.
§EASI scores and percent BSA affected were assessed in 77 and 114 participants for groups with and without AD-Pn, respectively, at 2 years.
¶PP-NRS scores were assessed in 107 and 176 participants for groups with and without AD-Pn, respectively, at baseline. Participants were asked for their average PP-NRS score in the previous 7 days.
††POEM scores were assessed in 57 and 101 participants for groups with and without AD-Pn, respectively, at 96 weeks.
‡‡DLQI scores were assessed in 75 and 106 participants for groups with and without AD-Pn, respectively, at 2 years.

AD-Pn, atopic dermatitis-associated prurigo nodules; BSA, body surface area; DLQI, Dermatology Life Quality Index; EASI 50, the proportion of participants with ≥50% improvement in Eczema Area and Severity Index over 24 months; EASI 75, the proportion of participants with ≥75% improvement in Eczema Area and Severity Index over 24 months; IGA, Investigator’s Global Assessment; Pn, prurigo nodules; POEM, Patient-Oriented Eczema Measure; PP-NRS, Peak Pruritus Numerical Rating Scale; SD, standard deviation.

## TABLE S3

Summary of baseline demographic and participant characteristics for participants who remained in the study through the observation period (without formal discontinuation).

|  | **AD-Pn at baseline** | | | | | |
| --- | --- | --- | --- | --- | --- | --- |
| **Characteristic** | **Yes (n = 93)** | | | **No (n = 146)** | | |
|  | **Overall (*n* = 93)** | **Hospital (*n* = 52)** | **Dermatology clinic (*n* = 41)** | **Overall (*n* = 146)** | **Hospital (*n* = 70)** | **Dermatology clinic (*n* = 76)** |
| Age, years | 36.4 ± 10.6 | 35.1 ± 10.0 | 38.1 ± 11.1 | 34.9 ± 10.6 | 33.8 ± 10.7 | 36.0 ± 10.4 |
| Sex, n (%) |  |  |  |  |  |  |
| Male | 68 (73.1) | 42 (80.8) | 26 (63.4) | 92 (63.0) | 31 (44.3) | 41 (53.9) |
| Female | 25 (26.9) | 10 (19.2) | 15 (36.6) | 54 (37.0) | 39 (55.7) | 35 (46.1) |
| Weight, kg | 63.6 ± 11.9 | 64.6 ± 12.9 | 62.5 ± 10.5 | 60.6 ± 12.0 | 58.2 ± 11.2 | 62.8 ± 12.4 |
| BMI, kg/m^2^ | 22.8 ± 4.00 | 23.0 ± 4.4 | 22.5 ± 3.5 | 22.5 ± 3.6 | 21.8 ± 3.2 | 23.1 ± 3.9 |
| Age of AD onset, years | 8.5 ± 10.8 | 10.8 ± 12.7 | 5.5 ± 6.7 | 8.0 ± 11.7 | 6.0 ± 10.2 | 9.8 ± 12.7 |
| AD disease duration, years | 28.0 ± 12.4 | 24.4 ± 11.5 | 32.6 ± 12.0 | 27.1 ± 11.8 | 28.0 ± 11.4 | 26.4 ± 12.1 |
| No. of visits for AD treatment in the previous year | 10.6 ± 12.8 | 11.6 ± 15.7 | 9.2 ± 7.4 | 8.6 ± 6.8 | 7.5 ± 5.3 | 9.7 ± 7.9 |
| IGA score | 3.4 ± 0.5 | 3.5 ± 0.5 | 3.3 ± 0.5 | 3.2 ± 0.4 | 3.4 ± 0.5 | 3.1 ± 0.2 |
| EASI score | 29.0 ± 15.7 | 31.6 ± 16.8 | 25.8 ± 13.7 | 24.9 ± 16.3 | 32.1 ± 18.9 | 18.2 ± 9.7 |
| BSA affected by AD, % | 54.5 ± 23.5 | 57.1 ± 25.2 | 51.2 ± 21.0 | 52.1 ± 24.8 | 62.7 ± 27.6 | 42.3 ± 17.1 |
| DLQI score | 8.1 ± 5.9 | 9.6 ± 6.7 | 6.3 ± 4.0 | 8.6 ± 7.1 | 11.8 ± 8.3 | 5.7 ± 4.1 |
| EQ-5D utility score | 0.7826 ± 0.1761 | 0.7416 ± 0.2011 | 0.8345 ± 0.1218 | 0.7825 ± 0.1981 | 0.6893 ± 0.2292 | 0.8670 ± 0.1118 |
| EQ-mobility (Q1): any problems, *n* (%) | 13 (14.0) | 9 (17.3) | 4 (9.8) | 23 (15.8) | 19 (27.5) | 4 (5.3) |
| EQ-self-care (Q2): any problems, *n* (%) | 18 (19.4) | 11 (21.2) | 7 (17.1) | 31 (21.2) | 19 (27.5) | 12 (15.8) |
| EQ-usual activity (Q3): any problems, *n* (%) | 41 (44.1) | 26 (50.0) | 15 (36.6) | 60 (41.1) | 42 (60.9) | 18 (23.7) |
| EQ-pain/discomfort (Q4): any problems, *n* (%) | 78 (83.9) | 44 (84.6) | 34 (82.9) | 114 (78.1) | 63 (91.3) | 51 (67.1) |
| EQ-anxiety/depression (Q5): any problems, *n* (%) | 42 (45.2) | 29 (55.8) | 13 (31.7) | 65 (44.8) | 45 (65.2) | 20 (26.3) |
| EQ-VAS | 60.0 ± 23.0 | 55.0 ± 23.7 | 65.7 ± 20.9 | 61.8 ± 22.4 | 53.1 ± 25.3 | 69.6 ± 16.1 |
| POEM score | 18.6 ± 6.3 | 19.8 ± 6.6 | 17.0 ± 5.5 | 16.1 ± 6.9 | 19.3 ± 6.6 | 13.2 ± 5.8 |
| Weekly average PP-NRS score† | 6.8 ± 2.0 | 7.1 ± 2.1 | 6.3 ± 1.8 | 6.5 ± 2.3 | 7.4 ± 2.1 | 5.7 ± 2.2 |
| In males | *n* = 67 | *n* = 42 | *n* = 25 | *n* = 72 | *n* = 31 | *n* = 41 |
|  | 6.6 ± 2.2 | 7.0 ± 2.3 | 6.1 ± 1.9 | 5.9 ± 2.2 | 7.0 ± 2.0 | 5.1 ± 2.1 |
| In females | *n* = 25 | *n* = 10 | *n* = 15 | *n* = 73 | *n* = 39 | *n* = 34 |
|  | 7.0 ± 1.5 | 7.6 ± 1.4 | 6.7 ± 1.5 | 7.1 ± 2.3 | 7.7 ± 2.2 | 6.4 ± 2.2 |

*Note:* Data are presented as mean ± SD unless stated otherwise.
†Participants were asked for their average PP-NRS score in the previous 7 days.

AD, atopic dermatitis; AD-Pn, atopic dermatitis-associated prurigo nodules; BMI, body mass index; BSA, body surface area; DLQI, Dermatology Life Quality Index; EASI, Eczema Area and Severity Index; EQ, EuroQOL; EQ-5D, EuroQOL group health questionnaire with five dimensions; IGA, Investigator’s Global Assessment; POEM, Patient-Oriented Eczema Measure; PP-NRS, Peak Pruritus Numerical Rating Scale; SD, standard deviation; VAS, visual analog scale.

## FIGURE S1

Participant flow diagram.

AD-Pn, atopic dermatitis-associated prurigo nodules; M, months; PRO, patient-reported outcomes. *^a^* Katoh, N *et al. J Dermatol* 2019; **46**: 290-300, *^b^* Katoh, N *et al. J Dermatol* 2022; **49**: 903-11, *^c^* Seventeen out of 19 were from a single site (clinic) that was closed in 2018 for reasons unrelated to the study. No participants from this site had baseline AD-Pn.

## FIGURE S2

Numbers of participants with atopic dermatitis-associated prurigo nodules over time in (A) males and (B) females with atopic dermatitis-associated prurigo nodules at baseline, and in (C) males and (D) females without prurigo nodules at baseline. Missing data applied to participants who had not dropped out.

AD-Pn, atopic dermatitis-associated prurigo nodules; Pn, prurigo nodules.

## FIGURE S3

**Respondents with any problems (%)**

**Q1: Mobility**

**Q2: Self-Care**

**Q3: Usual Activities**

**Q4: Pain/Discomfort**

**Q5: Anxiety/Depression**

AD-PN (+)

AD-PN (-)

Longitudinal changes in percentages of respondents with any problems in each EuroQOL group health questionnaire with five dimensions (EQ-5D) subdomain by baseline presence of atopic dermatitis-associated prurigo nodules.

## FIGURE S4

Change in biomarkers over time by baseline presence of atopic dermatitis-associated prurigo nodules for (A) serum thymus- and activation-regulated chemokine levels, (B) peripheral blood eosinophil count, (C) total immunoglobulin E levels, and (D) serum lactate dehydrogenase levels.

AD-Pn, atopic dermatitis-associated prurigo nodules; IgE, immunoglobulin E; LDH, lactate dehydrogenase; Pn, prurigo nodules; SD, standard deviation; TARC, thymus- and activation-regulated chemokine.

## FIGURE S5

Distribution of participants with atopic dermatitis-associated prurigo nodules at baseline according to severity classification Criteria II and III for (A) Eczema Area and Severity Index scores, (B) Peak Pruritus Numerical Rating Scale† scores, (C) Patient-Oriented Eczema Measure scores, and (D) Dermatitis Life Quality Index scores.

†Participants were asked for their average PP-NRS score in the previous 7 days. Italicized numbers indicate sample sizes for each group. Mean values are plotted above the error bars. AD, atopic dermatitis; AD-Pn, atopic dermatitis-associated prurigo nodules; DLQI, Dermatitis Life Quality Index; EASI, Eczema Area and Severity Index; Pn, prurigo nodules; POEM, Patient-Oriented Eczema Measure; PP-NRS, Peak Pruritus Numerical Rating Scale; SD, standard deviation.

## FIGURE S6

Distribution of participants with atopic dermatitis-associated prurigo nodules at baseline according to severity classification Criteria II and III for (A) serum thymus- and activation-regulated chemokine levels, (B) peripheral blood eosinophils, (C) total immunoglobulin E levels, and (D) serum lactate dehydrogenase levels.

Italicized numbers indicate sample sizes for each group. Mean values are plotted above the error bars. AD, atopic dermatitis; AD-Pn, atopic dermatitis-associated prurigo nodules; IgE, immunoglobulin E; LDH, lactate dehydrogenase; Pn, prurigo nodules; SD, standard deviation; TARC, thymus- and activation-regulated chemokine.

## FIGURE S7

Mean values for (A) percent affected body surface area, (B) EuroQOL questionnaire with 5 dimensions utility score, and (C) EuroQOL questionnaire with visual analog scale score among participants with “non-severe” and “severe” atopic dermatitis-associated prurigo nodules and participants without prurigo nodules.

Italicized numbers indicate sample sizes for each group. Mean values are plotted above the error bars. AD-Pn, atopic dermatitis-associated prurigo nodules; BSA, body surface area; EQ-5D, EuroQOL questionnaire with 5 dimensions; EQ-VAS, EuroQOL questionnaire with visual analog scale; Pn, prurigo nodules; SD, standard deviation.

## FIGURE S8

Baseline prurigo nodule status determined long-term outcomes of atopic dermatitis as assessed by mean ± standard deviation scores for (A) Eczema Area and Severity Index, and (B) Peak Pruritus-Numerical Rating Scale†, in participants without prurigo nodules and with “non-severe” and “severe” atopic dermatitis-associated prurigo nodules according to severity classification Criteria II and III.

†Participants were asked for their average PP-NRS score in the previous 7 days. AD-Pn, atopic dermatitis-associated prurigo nodules; EASI, Eczema Area and Severity Index; Pn, prurigo nodules; PP-NRS, Peak Pruritus Numerical Rating Scale; SD, standard deviation.

## FIGURE S9

Baseline prurigo nodule status determined long-term outcomes of atopic dermatitis as assessed by mean ± standard deviation scores for (A) Dermatitis Life Quality Index in participants without prurigo nodules and with “non-severe” and “severe” atopic dermatitis-associated prurigo nodules according to severity classification Criteria II and III over 24 months, and (B) Patient-Oriented Eczema Measure scores in participants without prurigo nodules and those with “non-severe” and “severe” atopic dermatitis-associated prurigo nodules according to severity classification Criteria II and III over 96 weeks.

AD-Pn, atopic dermatitis-associated prurigo nodules; DLQI, Dermatitis Life Quality Index; Pn, prurigo nodules; POEM, Patient-Oriented Eczema Measure; SD, standard deviation.

## FIGURE S10

Initial topical corticosteroid/topical calcineurin inhibitor strength index in participants without prurigo nodules and with “non-severe” and “severe” atopic dermatitis-associated prurigo nodules according to severity classification Criteria II and III.

Mean values are plotted above the error bars. AD-Pn, atopic dermatitis-associated prurigo nodules; AU, arbitrary units; Pn, prurigo nodules; SD, standard deviation; TCI, topical calcineurin inhibitor; TCS, topical corticosteroid.
